# Musculoskeletal decay and bone–muscle crosstalk during physical disuse: Mechanisms and integrated countermeasures

**DOI:** 10.1113/EP093790

**Published:** 2026-09-28

**Authors:** Julia Margarita Reyes, Guy Hajj‐Boutros, Dounia Rouabhia, José Morais, Felipe Salech, Gustavo Duque

**Affiliations:** ^1^ Geriatrics Section Clinical Hospital University of Chile Santiago Chile; ^2^ Research Institute McGill University Health Centre Montréal Québec Canada; ^3^ Division of Geriatric Medicine, Department of Medicine McGill University Montreal Québec Canada; ^4^ St Mary's Research Centre McGill University Montréal Québec Canada; ^5^ Center for Advanced Clinical Research (CICA) Clinical Hospital University of Chile Santiago Chile; ^6^ Simone & Edouard Schouela RUISSS McGill Centre of Excellence for Sustainable Health of Seniors (Schouela CEDurable) McGill University Montréal Québec Canada

**Keywords:** bed rest, bone, fat, muscle, osteoporosis, osteosarcopenia, sarcopenia

## Abstract

Older adults experience a markedly increased risk of falls and fractures following hospital discharge, largely due to the rapid musculoskeletal deterioration associated with bed rest. Although age‐related muscle and bone loss typically develops gradually, acute physical inactivity accelerates key biological processes underlying sarcopenia and osteoporosis, including muscle atrophy, anabolic resistance, increased bone resorption and reduced mechanical loading. Emerging evidence indicates that muscle, bone and adipose tissue communicate through interconnected biochemical pathways; however, the shared biological mechanisms underlying this crosstalk during acute unloading remain poorly understood. To review the effects of disuse on the muscle–bone–adipose axis, emphasizing disruption of inter‐tissue signalling pathways and evaluating integrated countermeasures, a narrative review was conducted using PubMed, Scopus and Web of Science, focusing on human bed rest and disuse studies, musculoskeletal crosstalk and multimodal interventions involving exercise and nutrition in older adults. Disuse rapidly disrupts musculoskeletal homeostasis by uncoupling bone turnover and inducing muscle anabolic resistance. These changes are accompanied by alterations in myokine and osteokine signalling, including increased myostatin, reduced irisin, elevated RANKL, bone and muscle steatosis, and sclerostin upregulation, further compromising muscle–bone interactions. Evidence supports multimodal interventions combining resistance exercise with optimized protein intake (1.2–1.5 g/kg/day) and nutritional supplements, including β‐hydroxy‐β‐methylbutyrate, vitamin D and creatine, to attenuate muscle and bone loss. Preventing disuse‐related musculoskeletal decline requires recognizing muscle and bone as an integrated functional unit. Tailored multimodal strategies may preserve musculoskeletal health and functional independence in hospitalized older adults, although implementation must consider frailty, comorbidities, safety and feasibility.

## Abstract (highlights)

- **What is the topic of this review?**
  This review examines how physical disuse and mechanical unloading disrupt the muscle–bone–adipose axis, accelerating musculoskeletal decay and osteosarcopenia in older adults.
- **What advances does it highlight?**
  This review highlights the mechanisms of inter‐tissue crosstalk failure and how disuse‐induced axis disruption impairs physical function in older adults. Additionally, it evaluates integrated exercise and nutritional countermeasures to mitigate osteosarcopenia and preserve functional independence.

## 1 INTRODUCTION

### 1.1 The clinical problem

In the months following hospitalization, older patients face an elevated risk of falls and fractures (Gardner et al., 2008; Qian et al., 2022). This is not a random occurrence but directly linked to the rapid and detrimental effects of bed rest on the musculoskeletal system. While the individual impacts of immobilization on muscle and bone have been studied for decades, current evidence suggests that these systems do not fail in isolation; they interact actively through complex biochemical and mechanical pathways (Figure 1).

**FIGURE 1:**
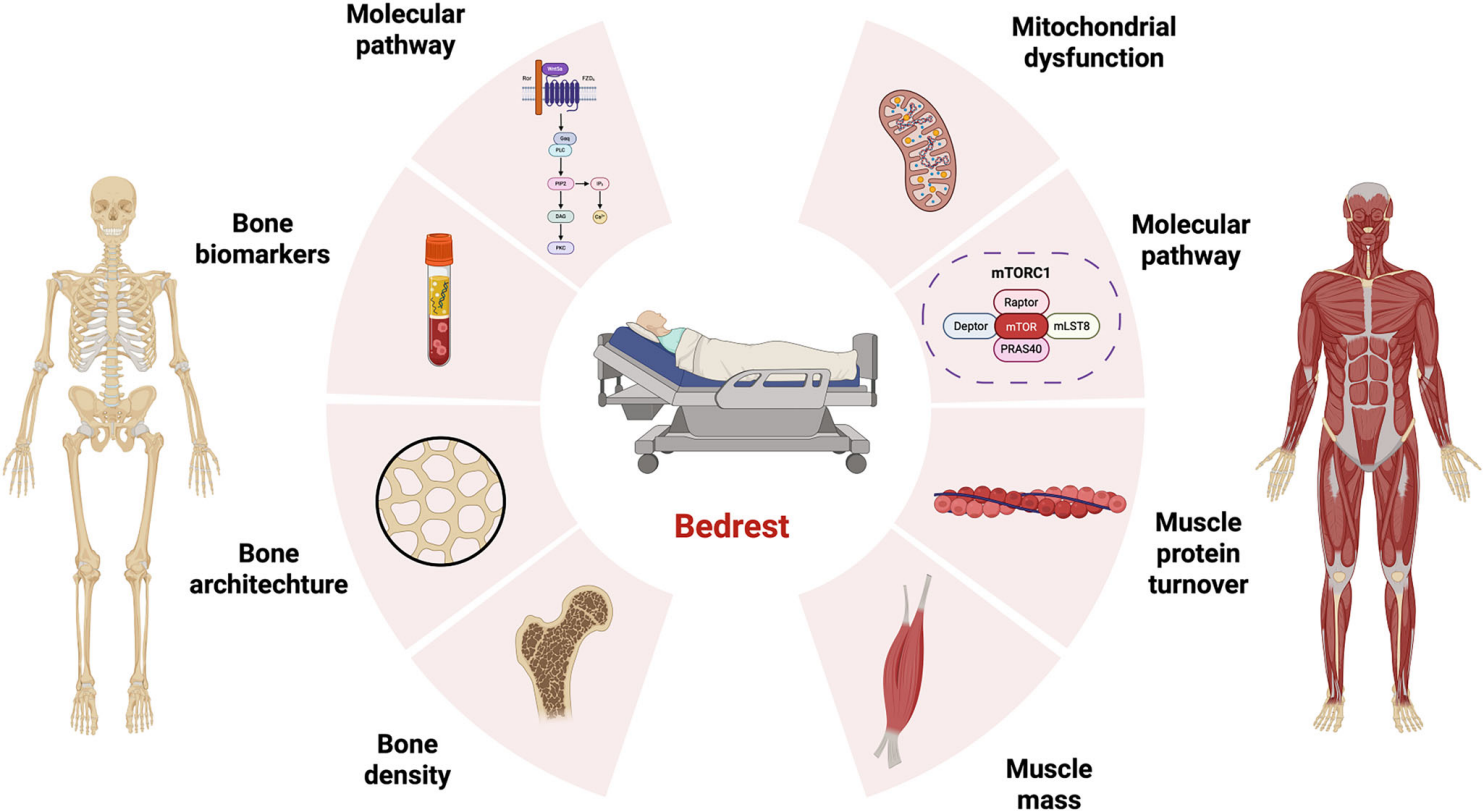
Bone and muscle markers altered during bedrest and addressed in this review.

Osteosarcopenia, defined as the concurrent deterioration of skeletal muscle mass and bone mineral density (BMD), represents a critical geriatric syndrome associated with frailty and mortality, with a prevalence estimated between 10% and 30% in community‐dwelling older adults (Hirschfeld et al., 2017). While age‐related decline is a gradual process, periods of acute physical inactivity, most notably bed rest during hospitalization, trigger a potent catabolic crisis, compressing years of physiological ageing into mere days (Hedge et al., 2022). However, a significant gap remains in the literature: while there is extensive research describing the effects of bed rest on muscle tissue, the literature focusing on how acute unloading affects the shared biological mechanisms between muscle and bone is scarce.

### 1.2 The physiology of disuse: An integrated decay

Evidence from experimental bed rest studies demonstrates that musculoskeletal deterioration occurs rapidly, is often profound and is highly site‐specific (Drummond et al., 2012; Kortebein et al., 2007; LeBlanc et al., 2007; Rittweger et al., 2009; Tanner et al., 2015). Therefore, throughout this review, findings from experimental bed rest studies are interpreted primarily as mechanistic evidence, with caution applied when extrapolating them to frail hospitalized older adults (Nunes et al., 2022). Historically, this has been viewed as two separate events. On one hand, muscle loss occurs disproportionately in weight‐bearing lower limbs; just 1 week of strict bed rest can induce a ∼1.6 kg reduction in lean mass, driven by a rapid suppression of myofibrillar protein synthesis and profound anabolic resistance to nutritional and mechanical stimuli (Drummond et al., 2012; Wall et al., 2014). Concurrently, bone tissue undergoes a process of ‘uncoupling’, where resorption is upregulated while formation remains suppressed, particularly at the femoral neck and lumbar spine (LeBlanc et al., 2007; Rittweger et al., 2009).

However, current paradigms shift away from these isolated views. This ‘double hit’ to the musculoskeletal system is not merely additive; it is the result of a disrupted muscle–bone–adipose axis. Mechanical unloading during bed rest triggers a ‘biochemical silence’ in which cross‐talk between myokines and osteokines is impaired, leading to a coordinated failure of functional capacity and explosive power (Rejc et al., 2018).

### 1.3 Objective

This narrative review synthesizes current evidence on the physiological consequences of bed rest and physical disuse on the muscle–bone axis, with particular attention to biological and functional features relevant to the osteosarcopenic phenotype. Rather than considering muscle and bone loss as isolated events, we critically examine how catabolic and unloading conditions disrupt shared mechanical, endocrine and molecular pathways involved in muscle–bone crosstalk. We also evaluate the evidence supporting integrated exercise and nutritional countermeasures, while addressing the translational challenges of applying findings from controlled bed rest studies to hospitalized older adults. Finally, we highlight key knowledge gaps, including sex‐specific responses, temporal patterns of adaptation and the need to validate predictive biomarkers that may guide future preventive and therapeutic strategies.

This review was informed by a comprehensive literature search conducted in PubMed, Scopus and Web of Science from database inception to May 2026. Searches included combinations of terms related to ‘bed rest’, ‘physical disuse’, ‘unloading’, ‘immobilization’, ‘muscle protein synthesis’, ‘muscle protein breakdown’, ‘bone turnover’, ‘sclerostin’, ‘RANKL’, ‘osteocalcin’, ‘myokines’, ‘osteokines’, ‘marrow adiposity’, ‘exercise countermeasures’ and ‘nutritional interventions’. Human experimental bed rest studies were prioritized, particularly those involving older adults or reporting clinically relevant functional outcomes, muscle or bone imaging, and biochemical markers of muscle and bone metabolism. When direct bed rest evidence was unavailable, studies using related disuse models, including limb immobilization or non‐weight‐bearing, were included to provide mechanistic context but were interpreted separately from whole‐body bed rest. Animal and cell studies were considered only when they addressed mechanisms not yet fully characterized in humans. Searches were conducted from database inception to January 2026. Evidence was interpreted according to study population, disuse model, duration of unloading, outcome type and relevance to clinical translation in older adults.

## 2 THE MUSCLE–BONE–ADIPOSE AXIS: CROSSTALK AND DISUSE

### 2.1 The biochemical dialogue: Myokines and osteokines

Bone and muscle share a functional unity rooted in embryogenesis, maintained not only by anatomical proximity but also by a continuous reciprocal exchange of mechanical and biochemical signals (Brotto & Johnson, 2014). This tightly integrated network is fundamental to musculoskeletal homeostasis; however, while the impact of age‐related decline is well‐recognized, the literature describing the disruption of these shared biological mechanisms specifically during acute bed rest remains scarce. Beyond mechanical loading, which drives remodelling through mechanotransduction (Duncan & Turner, 1995; Herrmann et al., 2020), both tissues function as active endocrine organs. The skeletal muscle secretome includes anabolic myokines such as insulin‐like growth factor 1 (IGF‐1), fibroblast growth factor 2 (FGF‐2), secreted protein acidic and rich in cysteine (SPARC), bone morphogenetic protein 1 (BMP‐1) and irisin, the latter being a critical driver of metabolic flexibility and bone health (Boström et al., 2012; Sui et al., 2024). Conversely, myostatin (growth differentiation factor 8, GDF‐8) and specific interleukins act as catabolic regulators that enhance bone resorption (Karsenty & Olson, 2016; Schnyder & Handschin, 2015; Wang et al., 2021). Reciprocally, bone‐derived osteokines modulate muscle physiology; specifically, osteocalcin signalling in myofibres is necessary for adaptation to exercise and ATP production (Mera et al., 2016), while prostaglandin E_2_ (PGE‐2) and Wnt3a further stimulate muscle growth. In contrast, under the ‘mechanical silence’ of bed rest, factors such as sclerostin, transforming growth factor β (TGF‐β) and receptor activator of nuclear factor κB ligand (RANKL) are upregulated, driving muscle degeneration and uncoupling bone turnover (Sui et al., 2024). To strengthen clinical translation, a clear distinction must be made between established pathways and emerging translational hypotheses within this endocrine network. Catabolic responses to unloading, including impaired anabolic signalling in skeletal muscle, myostatin‐related regulation of muscle atrophy, increased sclerostin and disruption of the RANKL–osteoprotegerin (OPG) axis, are supported by human disuse and bed rest studies (Bodine, 2013; Buehlmeier et al., 2017; Spatz et al., 2012; Yang et al., 2014). In contrast, the precise regulatory role of circulating anabolic mediators such as irisin, osteocalcin and FGF‐2 during acute human bed rest or clinical immobilization remains less established and should be considered an emerging translational hypothesis, largely supported by mechanistic, animal, exercise or broader muscle–bone crosstalk literature rather than direct clinical immobilization evidence (Karsenty & Olson, 2016; Kirk et al., 2020; G. B. Li et al., 2019). While rodent models demonstrate clear causal signalling for these factors, human evidence during acute bed rest is still predominantly suggestive and correlational, requiring further longitudinal validation.

### 2.2 The modulatory role of adipose tissue: Expanding the triad

Adipose tissue acts as a critical modulator of the musculoskeletal axis, effectively expanding the concept to a ‘bone–muscle–adipose triad’ that becomes particularly relevant during periods of inactivity. While adipose tissue is required for normal muscle development (Collins et al., 2022), the pathological accumulation of bone marrow adipose tissue (BMAT; Trudel et al., 2009) and intermuscular adipose tissue (IMAT; Prokopidis et al., 2026), both hallmarks of the bed rest model, introduces a potent source of pro‐inflammatory adipokines that disrupts the system. Within this triad, adiponectin exerts protective, anti‐proteolytic effects on muscle via receptors on osteoblasts and osteoclasts; consequently, its absence or suppression has been directly linked to accelerated muscle atrophy (Kirk et al., 2025; Krause et al., 2019). In contrast, leptin exhibits a paradoxical, context‐dependent role: while peripherally it promotes osteoblast activity and muscle growth, its central action via hypothalamic pathways inhibits bone formation (Chen & Yang, 2015; Kirk et al., 2025). This delicate balance is easily tilted toward a catabolic state when the mechanical and endocrine cues of the musculoskeletal unit are removed.

### 2.3 Disruption during disuse: The catabolic shift

Under conditions of mechanical unloading, this biochemical dialogue is fundamentally disrupted, precipitating a ‘catabolic shift’ that accelerates the clinical progression of osteosarcopenia. At the muscular level, disuse upregulates myostatin and interleukin (IL)‐6 while suppressing anabolic irisin, creating a signalling environment that favours systemic tissue degradation (Bosutti et al., 2008; Oranger et al., 2023; Sanesi et al., 2023; Wall et al., 2014). Simultaneously, the skeletal profile shifts toward resorption, characterized by reduced OPG and elevated sclerostin expression (Buehlmeier et al., 2017; Yang et al., 2014). Collectively, these molecular alterations reinforce a vicious cycle where muscle atrophy diminishes mechanical loading on bone, and compromised bone signalling further impairs muscle function. Understanding this synchronized failure is key to recognizing why bed rest induces such rapid functional decline, as the ‘mechanical silence’ effectively turns a once‐homeostatic triad into a coordinated catabolic engine.

## 3 THE MUSCLE UNIT UNDER STRESS: ANABOLIC RESISTANCE AND PROTEOLYSIS

### 3.1 Molecular signalling: The mTORC1 axis and proteolysis

At the molecular level, bed rest‐induced muscle atrophy is not an isolated event but a primary failure of the anabolic support within the musculoskeletal axis, orchestrated by the downregulation of the mechanistic target of rapamycin complex 1 (mTORC1) signalling pathway (Drummond et al., 2012). Disuse rapidly decreases phosphorylation of Akt (protein kinase B), a critical upstream regulator, thereby reducing activation of downstream anabolic targets including p70S6K and eukaryotic translation initiation factor 4E‐binding protein 1 (4E‐BP1) (Bodine et al., 2001; Kelleher et al., 2013). In older adults, this suppression is particularly pronounced; significant reductions in S6K1 and rpS6 phosphorylation are documented after only 5 days of bed rest, accompanied by the upregulation of mTORC1 inhibitors such as REDD1 and REDD2 (Tanner et al., 2015). Concurrently, catabolic pathways are upregulated to accelerate tissue decay. Markers of proteolysis, including AMP‐activated protein kinase α‐subunit (AMPKα) (an autophagy activator), the ubiquitin ligase atrogin‐1 (muscle atrophy F‐box, MAFBX) and the LC3II/I ratio (autophagy indicator), are elevated during bed rest in older adults compared to younger controls, suggesting a synergistic disruption of protein homeostasis (Tanner et al., 2015) that contributes to the rapid loss of muscle quality.

### 3.2 Dynamics of protein turnover: The primacy of impaired synthesis

The structural decline of muscle during disuse is driven by a net imbalance in protein turnover, characterized by a suppression of muscle protein synthesis (MPS) rather than a simple acceleration of breakdown. This defect is central to the osteosarcopenic phenotype, as the muscle fails to maintain the mass required for bone stimulation. Tracer studies consistently demonstrate that basal MPS declines sharply within days of inactivity. Shur et al. (2024) reported a 43% reduction in MPS after 3 days of bed rest; notably, although remobilization restored MPS in the exercised limb, the unloaded limb remained 35% below baseline, underscoring the necessity of mechanical loading for recovery. Similar magnitudes of suppression (27–50%) have been established across various disuse models, including 14‐day immobilization (Glover et al., 2008) and simulated microgravity (Ferrando et al., 1996). Furthermore, Symons et al. (2009) documented a 48.5% decrease in the fractional synthetic rate of the vastus lateralis after 21 days of bed rest, a deficit that was only effectively mitigated by interventions such as daily artificial gravity exposure. Importantly, this synthetic failure is not exclusive to younger cohorts; 10 days of strict experimental bed rest in healthy older adults has been demonstrated to induce a profound ∼30% reduction in muscle protein synthesis, directly driving a rapid loss of lower‐body lean mass (Kortebein et al., 2007).

Crucially, disuse also induces ‘anabolic resistance’, a phenomenon in which the muscle's sensitivity to nutrient and mechanical stimuli is blunted. Postprandial MPS can fall by approximately 50% within 5 days of unloading, rendering standard amino acid provision insufficient to maintain mass (Glover et al., 2008; Wall et al., 2014). A recent meta‐analysis of bed rest studies measuring MPS over timeframes ranging from hours to days confirms the negative impact of bed rest on MPS (Prokopidis et al., 2025). However, a critical translational caveat must be acknowledged: the studies compiled in such meta‐analyses are almost exclusively based on healthy volunteers under tightly controlled conditions (‘uncomplicated disuse’). In the clinical reality of a hospital ward, older patients rarely experience isolated mechanical unloading. Instead, their ‘complicated’ disuse is heavily compounded by systemic low‐grade inflammation, multimorbidity, malnutrition and polypharmacy (Nunes et al., 2022). These coexisting clinical factors exert cumulative, independent catabolic pressures that can drastically accelerate muscle proteolysis and further blunt protein synthesis, creating a severe ‘catabolic crisis’ that goes far beyond the physiological shifts observed in experimental laboratories.

### 3.3 Muscle protein breakdown and age‐related susceptibility

In contrast to the profound suppression of synthesis observed in clinical populations, muscle protein breakdown (MPB) appears to play a secondary role in early human disuse atrophy. This represents a significant divergence from animal models, where accelerated proteolysis often predominates as the primary driver of muscle decay (Nunes et al., 2022). In humans, however, isotopic measurements across protocols ranging from 4 to 21 days consistently show negligible alterations in the fractional breakdown rate of limb muscles (Brook et al., 2022; Symons et al., 2009). While Shur et al. (2024) noted a transient systemic reduction in MPB, the consensus remains that atrophy is primarily driven by a ‘synthetic failure’. This mechanism is particularly detrimental in older adults, who already exhibit baseline age‐related anabolic resistance (Dardevet et al., 2012). In this population, bed rest exacerbates the blunted anabolic response to amino acids by up to 35% compared to younger individuals, accelerating the progression of the osteosarcopenic phenotype and widening the gap in recovery potential (Biolo et al., 2017; Drummond et al., 2012; Tanner et al., 2015). Although some studies have assessed MPB during bed rest, quantifying breakdown remains methodologically challenging, and the relatively small number of available studies limits the strength of conclusions (O'Reilly et al., 2025).

### 3.4 Temporal dynamics of fibre‐type plasticity and myofibrillar alterations

Skeletal muscle demonstrates remarkable plasticity in response to both biological ageing and mechanical unloading, a process that directly influences the mechanical loading required for bone health. At baseline, ageing muscle is generally characterized by altered fibre‐type composition, reduced motor unit remodelling capacity and impaired contractile quality. During disuse, however, the time course of adaptation is heterogeneous. Early responses to short‐term bed rest appear to involve transcriptional and molecular remodelling before consistent changes are detectable at the protein or fibre‐type level. For example, short‐term bed rest may initiate changes in genes related to muscle phenotype and metabolism, while measurable shifts in myosin heavy chain protein expression or fibre‐type distribution may require longer unloading exposure or may become more evident during recovery (Monti et al., 2021). Therefore, evidence for a slow‐to‐fast phenotypic transition during short‐term bed rest should be interpreted cautiously and viewed as an evolving process rather than a completed adaptation. Longer‐duration unloading and disuse models suggest that this remodelling may eventually manifest as a reduction in the relative proportion of pure Type I fibres and an increase in hybrid fibre populations, such as 2AX and 1–2AX fibres (Borina et al., 2010; Brocca et al., 2012; Trappe et al., 2004). However, these adaptations do not necessarily follow the same trajectory as early changes in anabolic signalling, muscle protein synthesis or inflammatory biomarkers. Indeed, several molecular and circulating markers fluctuate over time during bed rest, indicating that single time‐point assessments may not fully capture the dynamic nature of disuse‐induced muscle remodelling.

Structural atrophy may also emerge rapidly, although its magnitude depends on the duration and model of unloading. A reduction of approximately 10% in muscle fibre cross‐sectional area has been reported after short periods of unloading in both young and older cohorts (Suetta et al., 2012). At the myofilament level, bed rest has been associated with reductions in myosin content across fibre types, while the myosin‐to‐actin ratio may remain relatively preserved (Borina et al., 2010). These structural and contractile alterations contribute to impaired muscle quality and reduced explosive power. From a clinical perspective, this early loss of power is particularly important because it may compromise the patient's ability to stabilize the body during sudden movements, thereby increasing the risk of falls after hospitalization.

### 3.5 Clinical assessment of disuse atrophy: Imaging modalities

Accurate quantification of disuse‐induced atrophy is critical for identifying patients at high risk of post‐hospitalization complications, yet it remains methodologically complex. Although dual‐energy X‐ray absorptiometry (DXA) is the clinical standard, its precision in the acute bed rest setting is often compromised by shifts in hydration status and food intake (Horber et al., 1992; Nana et al., 2012). Furthermore, DXA lacks the sensitivity of tomographic methods to detect the subtle, regional changes in muscle quality that precede functional failure in the musculoskeletal unit (Delmonico et al., 2008; Fuchs et al., 2023). Conversely, magnetic resonance imaging (MRI) offers superior volumetric resolution (Erlandson et al., 2016), providing a distinct advantage when assessing older populations where the preservation of specific muscle groups is vital for joint stability and bone loading.

This methodological divergence is underscored by recent findings in geriatric cohorts. In a 14‐day bed rest study of older adults, MRI successfully detected the protective effect of exercise on thigh muscle volume (Dulac et al., 2025), whereas DXA failed to identify significant differences in lean mass in the same cohort (Hajj‐Boutros et al., 2023). This discrepancy is less pronounced in younger, robust populations, in which studies report high concordance among MRI, computed tomography (CT) and DXA estimates of atrophy (Fuchs et al., 2025). However, for the hospitalized older adult, the higher spatial resolution of MRI is indispensable. It allows for the detection of localized adaptations and intramuscular changes that may be masked by technical variability in DXA, ensuring a more accurate assessment of the patient's ‘musculoskeletal reserve’ before discharge. To overcome the high operational costs and lack of portability often limiting MRI implementation at the bedside, musculoskeletal ultrasound has emerged as an accessible, non‐invasive alternative in disuse research (Franchi et al., 2018). Within this modality, conventional B‐mode ultrasound allows for the precise tracking of localized muscle thickness and architectural remodelling (Franchi et al., 2018), whereas panoramic (extended field‐of‐view) imaging enables the reliable assessment of muscle cross‐sectional area, demonstrating high concurrent validity and strong agreement with MRI‐derived cross‐sectional measurements (Ahtiainen et al., 2010; Franchi et al., 2018).

## 4 BONE MICROENVIRONMENT DURING UNLOADING: THE SCLEROSTIN–RANKL SURGE

### 4.1 Rapid activation of bone resorption: The first skeletal response

Mechanical unloading precipitates an immediate and sustained uncoupling of bone turnover, a process that mirrors the rapid anabolic resistance seen in muscle. This decline is driven primarily by the rapid activation of osteoclastic resorption. Markers of type I collagen degradation, specifically C‐terminal telopeptide (CTX) and N‐terminal telopeptide (NTX), rise within the first days of immobilization, signalling a fast‐acting skeletal response to the ‘mechanical silence’. Baecker et al. (2003) observed significant elevations in NTX (+28.7%) and CTX (+17.8%) by day 2 of bed rest; notably, while NTX peaked early, CTX remained elevated throughout the recovery phase.

Interestingly, this skeletal sensitivity to unloading appears conserved across the lifespan, representing a universal threat to musculoskeletal integrity. Buehlmeier et al. (2017) demonstrated that although younger men exhibit higher baseline turnover markers, the relative magnitude of the resorptive surge during bed rest is comparable to older adults. This suggests that the osteoclastic response to disuse is a fundamental physiological reflex that, in older patients, acts on a skeleton already thinned by age, thereby increasing the risk of fracture during initial attempts at mobilization.

### 4.2 The uncoupling of bone formation

Osteoblastic activity exhibits a refractory period or delayed suppression, establishing a ‘temporal uncoupling’ of remodelling that leaves the bone vulnerable. In stark contrast to the immediate resorptive surge, recovery is delayed. Markers of formation, including osteocalcin (OC), bone‐specific alkaline phosphatase (bAP) and type I procollagen peptides (PICP, P1NP), generally remain stable during short‐term disuse (Baecker et al., 2003). Even during prolonged immobilization, significant declines in these markers are often absent or delayed (Fiore et al., 1999; Zerwekh et al., 1998); for instance, Inoue et al. (2000) noted that PICP levels declined significantly only after day 50. This delay in bone formation is critical when considering the muscle–bone unit. While the muscle is rapidly losing mass and power (reducing mechanical stimuli), the bone remains in a state of high resorption without compensatory formation. However, a distinct anabolic rebound occurs upon remobilization. Multiple studies indicate that markers like P1NP and bAP surge significantly during the recovery period (Austermann et al., 2021; Kim et al., 2003; Yang et al., 2014). This highlights a key clinical window: the reactivation of osteoblastic function depends on restoring mechanical loading, underscoring the need for early integrated exercise interventions to ‘re‐couple’ the system and prevent progression of the osteosarcopenic phenotype.

### 4.3 Mechanotransduction pathways: The role of sclerostin and OPG

The molecular basis for uncoupling of bone turnover is largely governed by the Wnt signalling pathway, which acts as a bridge between mechanical loading and cellular response. Mechanical unloading stimulates osteocytes to upregulate sclerostin, a potent Wnt inhibitor that suppresses osteoblast differentiation (Choi & Robling, 2021). Belavý et al. (2016) reported an early rise in circulating sclerostin by day 12 of bed rest, which correlated with a subsequent decrease in bAP, effectively linking the ‘mechanical silence’ to impaired bone formation. This elevation is a consistent hallmark of disuse, appearing across various protocols from day 14 to day 28 (Frings‐Meuthen et al., 2013; Gaudio et al., 2010; Spatz et al., 2012).

Concurrently, the regulation of osteoclastogenesis is disrupted, further fracturing the muscle–bone balance. Yang et al. (2014) observed a 40% reduction in OPG, the decoy receptor that inhibits RANKL, during 60 days of bed rest, tipping the systemic balance toward bone resorption. Conversely, the recovery phase is characterized by a reversal of this ratio; increased OPG and reduced RANKL levels facilitate the restoration of bone mass (Buehlmeier et al., 2017). This molecular ‘switch’ highlights that the bone's endocrine environment is highly sensitive to the presence or absence of muscle‐generated tension.

### 4.4 Systemic mineral homeostasis

The uncoupling of bone turnover has profound systemic consequences that extend beyond the skeleton. Rapid and sustained bone resorption leads to significant hypercalciuria, detectable as early as day 1 (Baecker et al., 2003) and peaking around day 50 (Inoue et al., 2000). This massive efflux of calcium from the skeleton into the circulation suppresses parathyroid hormone (PTH) secretion during immobilization. This hormonal suppression typically rebounds significantly during the recovery phase (Hajj‐Boutros et al., 2023; Inoue et al., 2000), illustrating how local skeletal unloading triggers a systemic endocrine fluctuation that can complicate the metabolic management of the hospitalized older adult.

### 4.5 Clinical convergence: Ageing and disuse mechanisms

Physiological ageing and skeletal disuse share distinct yet convergent biological mechanisms that deleteriously affect bone integrity and functional independence. Age‐related bone loss is characterized by a structural phenotype of trabecular thinning, loss of connectivity and progressive cortical porosity (Chen et al., 2013). Disuse mimics and accelerates this trajectory; the absence of muscle‐derived mechanical stimuli, essential for regulating osteocyte mechanotransduction, precipitates rapid structural deterioration in both cortical and trabecular compartments (Klein‐Nulend et al., 2013).

Consequently, the superimposition of bed rest on the ageing skeleton results in a ‘double hit’ of compromised integrity and heightened fragility. This convergence explains why even short periods of hospitalization can lead to a catastrophic loss of skeletal strength, where the ‘mechanical silence’ of bed rest acts as a catalyst for the osteosarcopenic phenotype, ultimately manifesting as the high incidence of post‐discharge fractures.

### 4.6 Method‐dependent detection of bone loss: Beyond densitometry

Traditionally, monitoring skeletal changes during bed rest has relied on DXA. While DXA remains the clinical standard for measuring areal bone mineral density (aBMD), it fails to capture the microarchitectural determinants of bone strength, such as trabecular spacing and cortical porosity, which are the true drivers of fracture risk in the acutely immobilized patient (Sarfati et al., 2024; Sornay‐Rendu et al., 2017). In this context, high‐resolution peripheral quantitative computed tomography (HR‐pQCT) has emerged as a transformative tool. By quantifying volumetric BMD (vBMD) and specific indices like cortical thickness, HR‐pQCT facilitates finite element modelling to estimate failure load. This provides a functional measure of ‘bone competence’ that is unattainable by densitometry alone, allowing clinicians to identify the silent structural decay that occurs during bed rest (MacNeil & Boyd, 2007).

The detection of this decay is highly sensitive to the imaging modality and the duration of unloading. Using standard DXA, changes in aBMD are typically undetectable during short periods; for instance, McGrath et al. (2022) reported no significant alterations in BMD after 30 days of bed rest. However, when sensitivity is optimized, modest changes can be captured. For instance, Smith et al. (2009) observed significant declines in hip and trochanteric BMD following 21 days of unloading. In long‐term models, DXA consistently identifies pronounced, site‐specific losses in weight‐bearing regions. Shackelford et al. (2004) reported significant aBMD decreases after 17 weeks of bed rest in weight‐bearing sites, including the trochanter (−3.6%), femoral neck (−1.5%) and lumbar spine (−1.3%). Consistent with this, non‐weight‐bearing sites remain unaffected, as evidenced by a 0% change in the distal radius within the same cohort, which aligns with findings from other disuse studies (Watanabe et al., 2004; Zerwekh et al., 1998).

When assessed with advanced imaging (pQCT and HR‐pQCT), a more complex and concerning pattern of deterioration emerges. Armbrecht et al. (2011) and Beller et al. (2011) demonstrated that 60 days of bed rest induced significant cortical thinning and trabecular separation at the distal tibia. Notably, unlike DXA, these high‐resolution methods also detected significant structural deterioration at the distal radius, highlighting that the ‘mechanical silence’ of bed rest triggers a systemic skeletal response that has previously been overlooked.

Furthermore, advanced imaging has elucidated the critical phenomenon of ‘uncoupled recovery’, which accounts for the persistence of fracture risk after hospital discharge. Rittweger et al. (2010) found that the greatest bone loss at the distal tibia (−3.6%) occurred 14 days post‐remobilization, with up to 80% of mineral loss occurring during the early recovery phase rather than during the bed rest itself. Similarly, Kazakia et al. (2014) revealed that 6 weeks of non‐weight bearing induced a marked 16.1% increase in cortical porosity at the distal tibia, a structural defect that remained elevated throughout recovery even as other parameters improved. From a clinical perspective, this indicates that the ‘musculoskeletal unit’ is most vulnerable exactly when the patient begins to move again, reinforcing the need for integrated countermeasures that protect both muscle power and bone microarchitecture during this high‐risk transition.

### 4.7 The ‘recovery paradox’: Persistent and delayed deterioration

Skeletal deterioration does not cease immediately upon remobilization, creating a ‘recovery paradox’ where the risk of fracture may actually peak after the patient has left the bed. Rittweger et al. (2010) observed that up to 80% of diaphyseal and 40% of epiphyseal BMD loss occurred after the bed rest phase ended, identifying a ‘delayed deleterious effect’ that threatens the stability of the musculoskeletal unit during the initial stages of rehabilitation. In their 56‐day study, the greatest loss at the distal tibia (−3.6%) was recorded at day 14 of recovery, with epiphyseal restitution remaining incomplete even after 12 months. This persistent structural deficit is corroborated by Kazakia et al. (2014), who reported a marked increase in cortical porosity (+16.1%) at the ultradistal tibia that remained elevated throughout the recovery period. These findings underscore that the cortical compartment, the primary structural support for muscle‐generated tension, is particularly susceptible to persistent damage following disuse, explaining the high incidence of post‐hospitalization fractures.

### 4.8 Bone marrow adiposity: The osteoblast–adipocyte switch and osteoinflammation

Mechanical unloading in humans significantly accelerates bone marrow adiposity (BMA) accumulation. In long‐term bed rest models (60 days of head‐down tilt), lumbar vertebral fat fraction increased by +2.5% in healthy women (Trudel et al., 2009) and +3.6% in healthy men, exceeding the physiological rate by 36‐fold (Trudel et al., 2012). Remarkably, this 60‐day immobilization period accelerates normal bone marrow involution by approximately 4 years (Trudel et al., 2009). Crucially, this adipogenic drift is highly responsive to mechanical stimuli; while the accumulation persisted up to 1 year in female cohorts (Trudel et al., 2009), male subjects demonstrated a marked medullary reconversion during reambulation, with BMA completely returning to baseline values after 2 years (Liu et al., 2021).

At the cellular level, this disuse‐induced marrow adipose tissue (MAT) expansion is initiated by a lack of mechanical strain sensed by osteocytes, which autonomously upregulate sclerostin expression and elevate the RANKL/OPG ratio (Spatz et al., 2015). Sclerostin acts as a competitive antagonist that blocks canonical Wnt/β catenin signalling, the primary endogenous pathway inhibiting adipogenesis and promoting osteogenesis in mesenchymal stem cells (Bagchi & Macdougald, 2021). Histologically, while both ageing and disuse drive marrow adipocyte hypertrophy, prominent adipocyte hyperplasia represents the hallmark signature effect of immobilization on MAT (Zhou et al., 2020). Ultimately, this accelerated hyperplastic infiltration does not represent a passive substitution of tissue, as these accumulated MAT adipocytes actively contribute to enhanced osteoclastogenesis (Li et al., 2018). Distinct from non‐medullary adipocytes, marrow‐derived adipocytes actively secrete significant, regulated levels of IL‐6 alongside lower concentrations of IL‐1β and tumour necrosis factor‐α (Laharrague et al., 2000). This unique secretome establishes a chronic pro‐inflammatory microenvironment within the marrow niche, further impairing the bone's ability to respond to future anabolic signals and effectively locking the musculoskeletal unit in a sustained catabolic state.

## 5 FUNCTIONAL PERFORMANCE DECLINE: THE CLINICAL MANIFESTATION OF AXIS FAILURE

The molecular and structural alterations described above ultimately converge into rapid and clinically meaningful declines in physical function, often before substantial changes in muscle mass are detectable. Lower‐limb strength and power are particularly vulnerable to disuse and represent key predictors of mobility loss, falls and post‐hospitalization disability. In older adults, two weeks of bed rest have been shown to reduce leg extension power by approximately 15% and quadriceps force by approximately 13% (Elam et al., 2022; Pišot et al., 2016). Even shorter periods of mechanical unloading can be detrimental; after only 10 days of bed rest Kortebein et al. (2008) reported significant reductions in isotonic knee extensor strength (−13.2%) and stair‐climbing power (−14.1%). These deficits are clinically important because low knee extension strength has been associated with increased risk of permanent mobility limitation and higher mortality risk (English & Paddon‐Jones, 2010; Manini et al., 2007).

Disuse also compromises gait, balance and task‐specific functional capacity. Although habitual gait speed may remain relatively preserved in some studies, more sensitive parameters reveal early deterioration, including reductions in stride length (−9.9%) and 5‐min walk distance (−7.4%) in older adults following bed rest (Pišot et al., 2016; Rejc et al., 2018). Postural control is similarly affected, with a 45% increase in sway area observed after 2 weeks of unloading (Elam et al., 2022) indicating impaired balance and potentially increased fall risk. However, this finding should not be interpreted as evidence of a permanent impairment. Rather, it may reflect a delayed or incomplete postural control response during the early recovery period, highlighting the need for follow‐up assessments to determine the time course and reversibility of balance alterations after remobilization. Standard functional batteries, such as the Short Physical Performance Battery, may not always detect these acute changes with sufficient sensitivity (Kortebein et al., 2008). In contrast, task‐specific outcomes, such as stair ascent and descent, appear more responsive, with reported increases in stair ascent time (+21.1%) and descent time (+23.5%) after bed rest (Coker et al., 2015).

Together, these findings highlight an important function–structure discordance during disuse, whereby functional decline can precede or exceed measurable structural atrophy. Di Girolamo et al. (2021) reported that physical performance declined by approximately 3% per day during the first 5 days of bed rest, deteriorating faster than muscle mass.

## 6 FRACTURE RISK: STRUCTURAL, METABOLIC AND CLINICAL EVIDENCE

Although experimental bed rest studies are typically too short to observe fracture endpoints directly, the causal link between acute immobilization and heightened fracture risk is supported by a robust convergence of structural, metabolic and clinical evidence. This risk is the ultimate clinical manifestation of the ‘fractured’ muscle–bone dialogue previously discussed.

Mechanistically, high‐resolution imaging has bridged the gap between disuse and skeletal failure. Kazakia et al. (2014) demonstrated that unloading induces rapid cortical porosity and trabecular thinning, structural deficits that Sarfati et al. (2024) recently confirmed as powerful, independent predictors of incident fractures, often outperforming traditional densitometry. Metabolically, the uncoupled state of the skeleton provides further prognostic clarity. Eimori et al. (2016) provided critical evidence in long‐term bedridden patients, showing that bone turnover markers, specifically osteocalcin and NTX, remain chronically elevated at levels exceeding clinical cutoffs established for fracture prediction.

Clinically, this molecular and structural decay translates into catastrophic events. Observational studies in stroke survivors provide a ‘natural experiment’ that isolates disuse as a primary driver of bone failure: fracture risk increases dramatically, specifically on the paretic (immobile) side compared to the mobile limb (Ramnemark et al., 1998). Together, these lines of evidence underscore that the ‘mechanical silence’ of bed rest does not merely weaken the skeleton; it actively reconfigures its microarchitecture and metabolic set‐point toward a state of heightened fragility, directly predisposing the older adult to the cycle of falls and fractures that follows hospital discharge. These interconnected alterations are summarized in Figure 2, which illustrates how bed rest disrupts bone–muscle crosstalk and promotes adipose tissue accumulation, bone loss, muscle wasting and functional decline.

**FIGURE 2:**
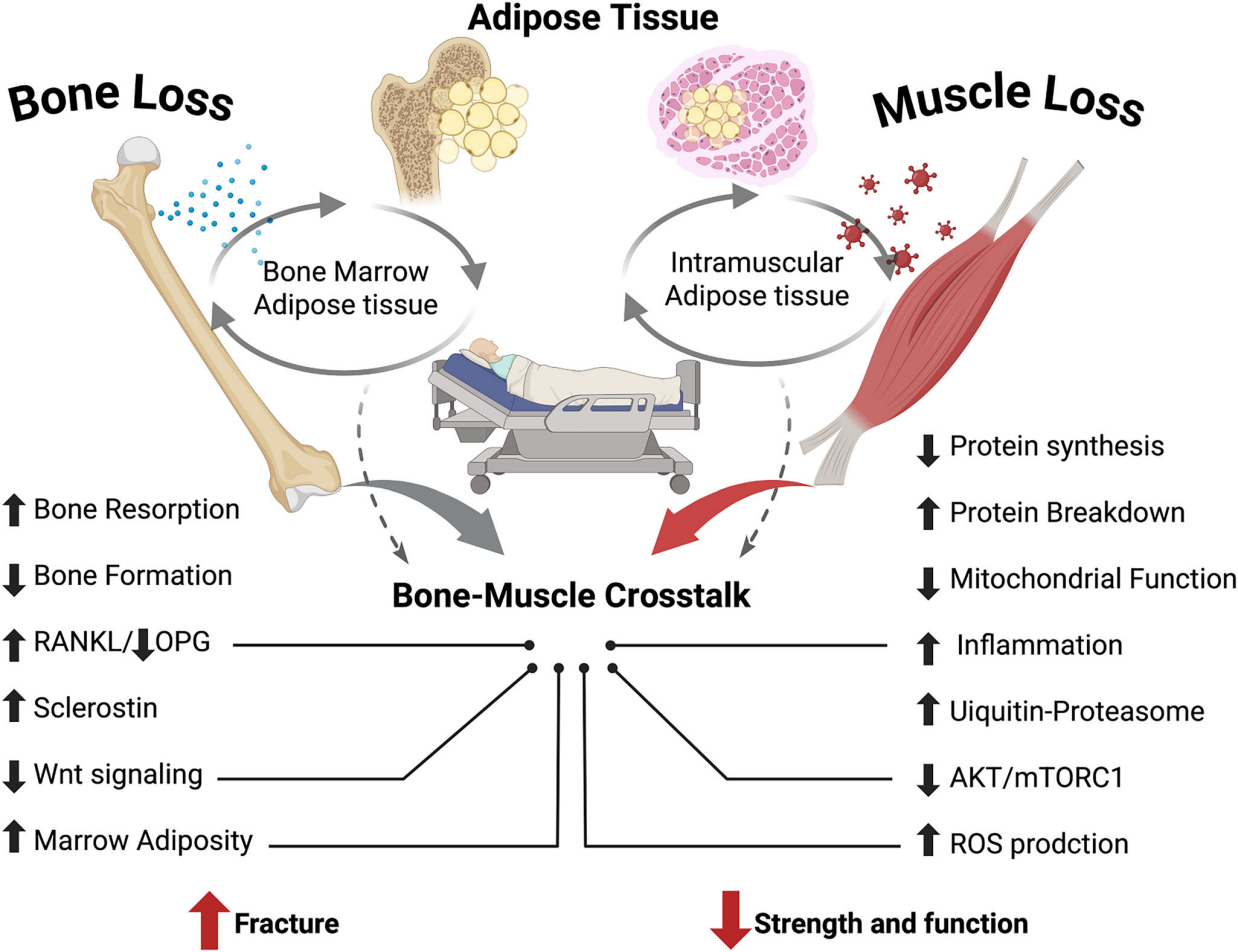
Osteosarcopenia and bedrest: Crosstalk mechanisms of muscle and bone loss.

## 7 INTEGRATED COUNTERMEASURES: RESTORING THE MUSCLE–BONE CROSS‐TALK

### 7.1 From passive care to active intervention: A paradigm shift

Historically, bed rest was viewed as a passive recovery phase. However, current evidence supports a paradigm shift toward active, multimodal interventions to mitigate the rapid, coordinated decay of the musculoskeletal unit and the development of bed rest‐induced osteosarcopenia. Exercise and nutritional support are no longer considered isolated treatments, but rather central and synergistic components of an integrated strategy aimed at preserving musculoskeletal reserve and restoring systemic crosstalk. Although implementation during immobilization can be logistically challenging, adapted protocols combining high‐load resistance training with aerobic exercise (including HIIT) have demonstrated clear efficacy in maintaining muscle function, bone integrity and metabolic health in experimental settings (Alkner & Tesch, 2004; Rittweger et al., 2009; Trappe et al., 2004). In parallel, nutritional support has evolved beyond simple caloric maintenance toward targeted approaches that address anabolic resistance, including protein optimization (1.2–1.5 g/kg/day) and the use of ergogenic aids such as β‐hydroxy‐β‐methylbutyrate (HMB), vitamin D and creatine, shifting clinical care from damage control to active preservation and improved recovery potential (Drummond et al., 2012; Paddon‐Jones et al., 2004; Pišot et al., 2016; Wall et al., 2014). Nevertheless, transitioning these multimodal strategies from experimental bed rest protocols to acute clinical settings involves a substantial translational barrier. While healthy volunteers demonstrate robust therapeutic responsiveness to physical and nutritional loading, hospitalized older patients present with systemic inflammation and multi‐pathology, clinical features that fundamentally heighten anabolic resistance and decrease exercise tolerance (Aarden et al., 2021). Furthermore, aggressive nutritional interventions often face clinical hurdles such as hospital‐associated anorexia or specialized diet restrictions, leading to mixed strength of evidence regarding their short‐term efficacy in overcoming anabolic muscle resistance and preventing acute bone loss (Dowling et al., 2024). Consequently, these countermeasures must be personalized and implemented not merely as performance interventions, but as targeted therapies designed to re‐establish the biological dialogue of the musculoskeletal unit under severe systemic stress. These countermeasures aim not only to protect individual tissues, but to preserve the systemic signalling necessary for long‐term musculoskeletal health.

### 7.2 Exercise interventions: The multimodal approach to systemic loading

Recent evidence highlights the superiority of multimodal exercise protocols over single‐modality interventions, particularly for the older patient whose muscle–bone axis is most fragile. Dulac et al. (2025) investigated a 14‐day bed rest protocol in adults aged 55–65, employing a daily hour of supine exercise that integrated high‐intensity interval training (HIIT), continuous aerobic training and lower‐body resistance work. The results were compelling: while controls exhibited significant, persistent atrophy in the upper quadriceps, the multimodal group successfully maintained muscle volume throughout both the bed rest and recovery phase.

Beyond structural preservation, specific modalities appear to protect the cellular bioenergetics necessary for tissue communication. Resistive vibration exercise (RVE) has been shown to attenuate the decline in mitochondrial respiration following 21 days of bed rest (Kenny et al., 2017), while flywheel resistance exercise effectively preserved mitochondrial content during an 84‐day intervention (Irimia et al., 2017). Regarding volume retention, Miokovic et al. (2014) demonstrated that even short‐duration resistive protocols (5–6 min), performed with or without whole‐body vibration, significantly blunted lower limb atrophy. These findings, supported by systematic reviews (González‐Rocha et al., 2022) confirm that concurrent resistance and aerobic training is the most comprehensive countermeasure to prevent the mechanical and metabolic uncoupling of the system. However, translating these protocols into acute hospital wards faces major challenges in practical feasibility and patient adherence. High‐intensity interventions are often unfeasible due to severe fatigue, delirium or understaffing. Furthermore, medical contraindications, such as cardiovascular instability or recent surgery, restrict participation. Therefore, clinical deployment demands a pragmatic, tiered approach prioritizing early, low‐intensity mobilization.

### 7.3 Nutritional countermeasures: Optimizing the molecular environment

Nutritional support is a cornerstone in managing osteosarcopenia, specifically designed to counteract the anabolic resistance induced by inactivity and inflammation. While mechanical loading is the primary stimulus, nutritional interventions modulate the underlying molecular pathways of protein synthesis and bone turnover, ‘re‐sensitizing’ the tissues to anabolic signals. However, the therapeutic efficacy of these countermeasures is highly dependent on the clinical context (Table 1), varying dramatically between models of uncomplicated disuse, such as healthy experimental bed rest, and complicated disuse, which is characterized by acute or chronic systemic illness (Nunes et al., 2022). Critically, much of the evidence for these interventions derives from ambulatory or frail cohorts rather than strict experimental bed rest protocols. A clear distinction is therefore required between compounds directly validated in human mechanical unloading settings and those relying on general geriatric extrapolation. Supplement efficacy in disuse atrophy is summarized in Table 1.

**TABLE 1:** Supplement efficacy in disuse atrophy: Primary mechanisms and key clinical endpoints.

Supplement/nutrient	Primary mechanism	Study population and evidence context	Clinical impact (measurable values)
**HMB**	Anti‐catabolic; inhibits ubiquitin‐proteasome pathway	Healthy older adults (experimental bed rest; moderate‐to‐high evidence)	Preserves ∼1 kg of leg lean mass over 10 days of bed rest compared to placebo (Deutz et al., 2013). Prevents the decline in strength during rehabilitation
**Creatine**	Increases phosphocreatine stores and myogenic factors	Community‐dwelling older adults (combined with training; high evidence)	Increases lean tissue mass by +1.37 kg and appendicular strength (SMD 0.29) in older adults. Boosts bone mineral content by +3.2% in upper limbs when combined with resistance training (Chilibeck et al., 2015)
**Protein**	Stimulates intracellular anabolic signalling to overcome resistance	Healthy older adults (short‐term immobilization; high evidence)	Intake must exceed 1.2–1.5 g/kg/day to stimulate synthesis (Kirk et al., 2021). Lower doses (<0.8 g/kg) fail to attenuate atrophy (∼1.5% CSA loss) during short‐term disuse (English et al., 2010)
**Vitamin D**	Calcium homeostasis and Type II fibre maintenance	Vitamin‐deficient clinical geriatric cohorts (high evidence)	Doses of 800–1000 IU/day reduce fall risk by ∼15%–20% and osteoporotic fracture risk in vitamin‐deficient older adults (Kirk et al., 2021)
**Omega‐3**	Anti‐inflammatory; sensitizes muscle to anabolism	Healthy older adults (metabolic ward/experimental; moderate evidence)	Daily high‐dose (3–4 g) doubles muscle membrane EPA/DHA content and significantly restores anabolic sensitivity to amino acids (Bird et al., 2021; Orchard et al., 2012; Smith et al., 2011)

***Note***: Evidence levels are classified based on the clinical study design hierarchy (GRADE working group criteria): high evidence denotes data derived from large‐scale randomized controlled trials, meta‐analyses or international consensus guidelines; moderate evidence denotes data from smaller metabolic ward trials, mechanistic studies or well‐controlled experimental disuse models with limited sample sizes. CSA, cross‐sectional area; DHA, docosahexaenoic acid; EPA, eicosapentaenoic acid; HMB, β‐hydroxy‐β‐methylbutyrate; SMD, standardized mean difference.

#### 7.3.1 Micronutrients and anti‐inflammatory agents

Vitamin D and calcium remain the standard of care to ensure adequate substrate for mineralization and contractile function, particularly given their high deficiency rates in older populations (Kirk et al., 2021). Additionally, omega‐3 fatty acids (specifically eicosapentaenoic acid [EPA] and docosahexaenoic acid [DHA]) have emerged as potential countermeasures of sarcopenia and osteoporosis by dampening the systemic inflammation often seen during mechanical unloading; they may sensitize skeletal muscle to anabolic stimuli (Bird et al., 2021; Orchard et al., 2012; Smith et al., 2011).

#### 7.3.2 Protein intake and quality

To overcome age‐related anabolic resistance during bed rest, protein intake must be elevated to 1.2–1.5 g/kg/day (English & Paddon‐Jones, 2010; Kirk et al., 2021). Controlled human bed rest investigations confirm that essential amino acid and high‐quality protein supplementation can effectively mitigate the decline in myofibrillar protein synthesis under conditions of mechanical unloading (Paddon‐Jones et al., 2004). Protein quality is equally critical; sources rich in leucine are particularly effective, as this amino acid acts as a direct signalling molecule to activate the mTOR pathway, a crucial regulator of protein synthesis in the face of disuse (Cholewa et al., 2017).

#### 7.3.3 Ergogenic aids and novel targets

Substantial evidence supports the use of targeted aids such as HMB, a leucine metabolite that acts as a potent anti‐catabolic agent capable of preserving lean mass during bed rest in older adults (Deutz et al., 2013; Standley et al., 2017). Concurrently, creatine monohydrate supplementation has been shown to enhance both muscle mass and bone geometry, likely through the upregulation of myogenic transcription factors and increased metabolic activity in both myocytes and osteoblasts (Candow et al., 2022; Chilibeck et al., 2015). Finally, the emerging ‘gut–muscle–bone axis’ represents a novel therapeutic frontier, where prebiotics and probiotics may modulate the systemic environment to actively mitigate muscle wasting (Buigues et al., 2016; Prokopidis et al., 2023; Ticinesi et al., 2017). Nevertheless, the overall evidence remains mixed. While experimental models support the short‐term anti‐catabolic efficacy of compounds like HMB, definitive clinical trials evaluating their capacity to reduce hard clinical outcomes, such as post‐discharge fractures or functional dependency in acute geriatric cohorts, are still limited.

## 8 CURRENT LIMITATIONS AND FUTURE DIRECTIONS

### 8.1 Methodological constraints: Sample size and demographics

Despite significant progress in characterizing disuse physiology, the literature remains constrained by methodological limitations that hinder clinical translation. The majority of bed rest studies rely on small sample sizes (often *n* < 20) due to the prohibitive costs and logistical demands of conducting long‐term protocols in specialized facilities. Consequently, statistical power is frequently insufficient to detect subtle interaction effects, particularly when stratifying by biological variables. Furthermore, a critical demographic gap persists: while older adults represent the population most vulnerable to hospitalization‐associated osteosarcopenia, most experimental data are derived from healthy young males. This under‐representation fails to account for age‐related anabolic resistance, comorbidities and polypharmacy, thereby limiting the external validity of findings to real‐world geriatric settings where the ‘catabolic crisis’ is most severe.

### 8.2 The knowledge gap: Sex differences and biomarkers

The influence of biological sex on disuse atrophy remains a significant frontier. Given the sexual dimorphism in bone turnover, characterized by accelerated post‐menopausal resorption in women, there is a pressing need for sex‐specific analyses to determine if current countermeasures are equally effective across sexes. Additionally, while the concept of muscle–bone crosstalk is theoretically established, the temporal dynamics of specific signalling mediators (e.g., osteocalcin, fibroblast growth factor 23, irisin) during acute immobilization require further elucidation. Identifying the ‘biochemical signature’ of this axis failure is essential for developing predictive biomarkers to identify patients at the highest risk of post‐discharge complications.

### 8.3 Strategic imperatives

Coordinated efforts between space agencies, academic centres and clinical researchers could facilitate the creation of large, harmonized datasets, standardizing protocols for advanced imaging (HR‐pQCT) and functional assessment. Finally, the ultimate challenge lies in clinical translation. Validating exercise strategies in acutely ill, hospitalized older adults is critical. Future research must focus on adapting intensity and delivery methods, such as bedside ergometry or neuromuscular electrical stimulation, integrated within multidisciplinary care models to ensure feasibility, safety and adherence in the complex hospital environment.

## 9 CONCLUSION

Bed rest‐induced osteosarcopenia represents a critical catabolic crisis, particularly in older patients, characterized not only by independent tissue loss, but also by a profound disruption of the muscle–bone–adipose axis. This narrative review highlights that the clinical problem of falls and fractures in the months following hospital discharge is rooted in a ‘biochemical silence’ and a mechanical unloading that fractures the systemic communication between myokines and osteokines. As emphasized throughout this synthesis, while the effects on muscle are widely recognized, the coordinated decay of the musculoskeletal unit, driven by mechanisms such as sclerostin upregulation, RANKL surge and marrow fat infiltration, is where the most significant knowledge gaps and therapeutic opportunities lie.

Addressing this challenge requires a definitive paradigm shift: moving away from passive care toward early, multimodal and integrated interventions. The combination of high‐load resistance training while still at the hospital combined with precision nutritional support, specifically protein optimization, HMB and vitamin D, has proven synergistic in mitigating the effects of disuse. However, these countermeasures must be implemented not as isolated treatments for ‘weak muscles’ or ‘thin bones’, but as a systemic strategy to restore the endocrine dialogue of the musculoskeletal unit.

Ultimately, recognizing the shared biological pathways of disuse is essential to improving clinical outcomes. Future research should prioritize the validation of dual‐purpose biomarkers and the optimization of transitional care protocols to preserve functional independence and reduce the devastating burden of post‐hospitalization musculoskeletal failure.

## AUTHOR CONTRIBUTIONS

Julia Margarita Reyes, Guy Hajj‐Boutros and Gustavo Duque conceptualized the idea. Julia Margarita Reyes and Guy Hajj‐Boutros performed the literature search and summarized the evidence. All authors wrote and revised the preliminary and final versions of the manuscript. All authors have read and approved the final version of this manuscript and agree to be accountable for all aspects of the work in ensuring that questions related to the accuracy or integrity of any part of the work are appropriately investigated and resolved. All persons designated as authors qualify for authorship, and all those who qualify for authorship are listed.

## CONFLICT OF INTEREST

None declared.

## FUNDING INFORMATION

None.

## GENERATIVE AI STATEMENT

Gemini was used for language translation.

## Data Availability

No datasets were generated or analysed during the current study.

## References

[eph70468-bib-0001] Aarden, J. J. , Reijnierse, E. M. , van der Schaaf, M. , van der Esch, M. , Reichardt, L. A. , van Seben, R. , Bosch, J. A. , Twisk, J. W. R. , Maier, A. B. , Engelbert, R. H. H. , Buurman, B. M. , Kuper, I. , de Jonghe, A. , Kamper, A. , Posthuma, N. , Brendel, N. , & Wold, J. (2021). Longitudinal changes in muscle mass, muscle strength, and physical performance in acutely hospitalized older adults. Journal of the American Medical Directors Association, 22(4), 839–845.e1. 10.1016/j.jamda.2020.12.006 33428891

[eph70468-bib-0002] Ahtiainen, J. P. , Hoffren, M. , Hulmi, J. J. , Pietikäinen, M. , Mero, A. A. , Avela, J. , & Häkkinen, K. (2010). Panoramic ultrasonography is a valid method to measure changes in skeletal muscle cross‐sectional area. European Journal of Applied Physiology, 108(2), 273–279. 10.1007/s00421-009-1211-6 19777252

[eph70468-bib-0003] Alkner, B. A. , & Tesch, P. A. (2004). Efficacy of a gravity‐independent resistance exercise device as a countermeasure to muscle atrophy during 29‐day bed rest. Acta Physiologica Scandinavica, 181(3), 345–357. 10.1111/j.1365-201X.2004.01293.x 15196095

[eph70468-bib-0004] Armbrecht, G. , Belavý, D. L. , Backström, M. , Beller, G. , Alexandre, C. , Rizzoli, R. , & Felsenberg, D. (2011). Trabecular and cortical bone density and architecture in women after 60 days of bed rest using high‐resolution pQCT: WISE 2005. Journal of Bone and Mineral Research, 26(10), 2399–2410. 10.1002/jbmr.482 21812030

[eph70468-bib-0005] Austermann, K. , Baecker, N. , Zwart, S. R. , Fimmers, R. , Frippiat, J. P. , Stehle, P. , Smith, S. M. , & Heer, M. (2021). Antioxidant supplementation does not affect bone turnover markers during 60 days of 6° head‐down tilt bed rest: Results from an exploratory randomized controlled trial. Journal of Nutrition, 151(6), 1527–1538. 10.1093/jn/nxab036 33831949

[eph70468-bib-0006] Baecker, N. , Tomic, A. , Mika, C. , Gotzmann, A. , Platen, P. , Gerzer, R. , & Heer, M. (2003). Bone resorption is induced on the second day of bed rest: Results of a controlled crossover trial. Journal of Applied Physiology, 95(3), 977–982. 10.1152/japplphysiol.00264.2003 12909597

[eph70468-bib-0007] Bagchi, D. P. , & Macdougald, O. A. (2021). Wnt Signaling: From mesenchymal cell fate to lipogenesis and other mature adipocyte functions. Diabetes, 70(7), 1419–1430. 10.2337/dbi20-0015 PMC8336005 34155042

[eph70468-bib-0008] Belavý, D. L. , Baecker, N. , Armbrecht, G. , Beller, G. , Buehlmeier, J. , Frings‐Meuthen, P. , Rittweger, J. , Roth, H. J. , Heer, M. , & Felsenberg, D. (2016). Serum sclerostin and DKK1 in relation to exercise against bone loss in experimental bed rest. Journal of Bone and Mineral Metabolism, 34(3), 354–365. 10.1007/s00774-015-0681-3 26056021

[eph70468-bib-0009] Beller, G. , Belavỳ, D. L. , Sun, L. , Armbrecht, G. , Alexandre, C. , & Felsenberg, D. (2011). WISE‐2005: Bed‐rest induced changes in bone mineral density in women during 60 days simulated microgravity. Bone, 49(4), 858–866. 10.1016/j.bone.2011.06.021 21723970

[eph70468-bib-0010] Biolo, G. , Pišot, R. , Mazzucco, S. , Di Girolamo, F. G. , Situlin, R. , Lazzer, S. , Grassi, B. , Reggiani, C. , Passaro, A. , Rittweger, J. , Gasparini, M. , Šimunič, B. , & Narici, M. (2017). Anabolic resistance assessed by oral stable isotope ingestion following bed rest in young and older adult volunteers: Relationships with changes in muscle mass. Clinical Nutrition, 36(5), 1420–1426. 10.1016/j.clnu.2016.09.019 27742138

[eph70468-bib-0011] Bird, J. K. , Troesch, B. , Warnke, I. , & Calder, P. C. (2021). The effect of long chain omega‐3 polyunsaturated fatty acids on muscle mass and function in sarcopenia: A scoping systematic review and meta‐analysis. Clinical Nutrition ESPEN, 46, 73–86. 10.1016/j.clnesp.2021.10.011 34857251

[eph70468-bib-0012] Bodine, S. C. (2013). Disuse‐induced muscle wasting. International Journal of Biochemistry and Cell Biology, 45(10), 2200–2208. 10.1016/j.biocel.2013.06.011 PMC3856924 23800384

[eph70468-bib-0013] Bodine, S. C. , Stitt, T. N. , Gonzalez, M. , Kline, W. O. , Stover, G. L. , Bauerlein, R. , Zlotchenko, E. , Scrimgeour, A. , Lawrence, J. C. , Glass, D. J. , & Yancopoulos, G. D. (2001). Akt/mTOR pathway is a crucial regulator of skeletal muscle hypertrophy and can prevent muscle atrophy in vivo. Nature Cell Biology, 3(11), 1014–1019. 10.1038/ncb1101-1014 11715023

[eph70468-bib-0014] Borina, E. , Pellegrino, M. A. , D'Antona, G. , & Bottinelli, R. (2010). Myosin and actin content of human skeletal muscle fibers following 35 days bed rest. Scandinavian Journal of Medicine and Science in Sports, 20(1), 65–73. 10.1111/j.1600-0838.2009.01029.x 19883388

[eph70468-bib-0015] Boström, P. , Wu, J. , Jedrychowski, M. P. , Korde, A. , Ye, L. , Lo, J. C. , Rasbach, K. A. , Boström, E. A. , Choi, J. H. , Long, J. Z. , Kajimura, S. , Zingaretti, M. C. , Vind, B. F. , Tu, H. , Cinti, S. , Højlund, K. , Gygi, S. P. , & Spiegelman, B. M. (2012). A PGC1‐α‐dependent myokine that drives brown‐fat‐like development of white fat and thermogenesis. Nature, 481(7382), 463–468. 10.1038/nature10777 PMC3522098 22237023

[eph70468-bib-0016] Bosutti, A. , Malaponte, G. , Zanetti, M. , Castellino, P. , Heer, M. , Guarnieri, G. , & Biolo, G. (2008). Calorie restriction modulates inactivity‐induced changes in the inflammatory markers C‐reactive protein and pentraxin‐3. Journal of Clinical Endocrinology and Metabolism, 93(8), 3226–3229. 10.1210/jc.2007-1684 18492758

[eph70468-bib-0017] Brocca, L. , Cannavino, J. , Coletto, L. , Biolo, G. , Sandri, M. , Bottinelli, R. , & Pellegrino, M. A. (2012). The time course of the adaptations of human muscle proteome to bed rest and the underlying mechanisms. The Journal of Physiology, 590(20), 5211–5230. 10.1113/jphysiol.2012.240267 PMC3497573 22848045

[eph70468-bib-0018] Brook, M. S. , Stokes, T. , Gorissen, S. H. M. , Bass, J. J. , McGlory, C. , Cegielski, J. , Wilkinson, D. J. , Phillips, B. E. , Smith, K. , Phillips, S. M. , & Atherton, P. J. (2022). Declines in muscle protein synthesis account for short‐term muscle disuse atrophy in humans in the absence of increased muscle protein breakdown. Journal of Cachexia, Sarcopenia and Muscle, 13(4), 2005–2016. 10.1002/jcsm.13005 PMC9397550 35606155

[eph70468-bib-0121] Brotto, M. , & Johnson, M. L. (2014). Endocrine crosstalk between muscle and bone. Current Osteoporosis Reports, 12(2), 135–141. 10.1007/s11914-014-0209-0 PMC4374433 24667990

[eph70468-bib-0019] Buehlmeier, J. , Frings‐Meuthen, P. , Mohorko, N. , Lau, P. , Mazzucco, S. , Ferretti, J. L. , Biolo, G. , Pisot, R. , Simunic, B. , & Rittweger, J. (2017). Markers of bone metabolism during 14 days of bed rest in young and older men. Journal of Musculoskeletal & Neuronal Interactions, 17(1), 399–408. PMC5383768 28250244

[eph70468-bib-0020] Buigues, C. , Fernández‐Garrido, J. , Pruimboom, L. , Hoogland, A. J. , Navarro‐Martínez, R. , Martínez‐Martínez, M. , Verdejo, Y. , Carmen Mascarós, M. , Peris, C. , & Cauli, O. (2016). Effect of a prebiotic formulation on frailty syndrome: A randomized, double‐blind clinical trial. International Journal of Molecular Sciences, 17(6), 932. 10.3390/ijms17060932 PMC4926465 27314331

[eph70468-bib-0021] Candow, D. G. , Chilibeck, P. D. , Forbes, S. C. , Fairman, C. M. , Gualano, B. , & Roschel, H. (2022). Creatine supplementation for older adults: Focus on sarcopenia, osteoporosis, frailty and Cachexia. Bone, 162, 116467. 10.1016/j.bone.2022.116467 35688360

[eph70468-bib-0022] Chen, H. , Zhou, X. , Fujita, H. , Onozuka, M. , & Kubo, K. Y. (2013). Age‐related changes in trabecular and cortical bone microstructure. International Journal of Endocrinology, 2013, 213234. 10.1155/2013/213234 PMC3614119 23573086

[eph70468-bib-0023] Chen, X. X. , & Yang, T. (2015). Roles of leptin in bone metabolism and bone diseases. Journal of Bone and Mineral Metabolism, 33(5), 474–485. 10.1007/s00774-014-0569-7 25777984

[eph70468-bib-0024] Chilibeck, P. D. , Candow, D. G. , Landeryou, T. , Kaviani, M. , & Paus‐Jenssen, L. (2015). Effects of creatine and resistance training on bone health in postmenopausal women. Medicine and Science in Sports and Exercise, 47(8), 1587–1595. 10.1249/MSS.0000000000000571 25386713

[eph70468-bib-0025] Choi, R. B. , & Robling, A. G. (2021). The Wnt pathway: An important control mechanism in bone's response to mechanical loading. Bone, 153, 116087. 10.1016/j.bone.2021.116087 PMC8478810 34271473

[eph70468-bib-0026] Cholewa, J. M. , Dardevet, D. , Lima‐Soares, F. , de Araújo Pessôa, K. , Oliveira, P. H. , dos Santos Pinho, J. R. , Nicastro, H. , Xia, Z. , Cabido, C. E. T. , & Zanchi, N. E. (2017). Dietary proteins and amino acids in the control of the muscle mass during immobilization and aging: Role of the MPS response. Amino Acids, 49(5), 811–820. 10.1007/s00726-017-2390-9 28175999

[eph70468-bib-0027] Coker, R. H. , Hays, N. P. , Williams, R. H. , Wolfe, R. R. , & Evans, W. J. (2015). Bed rest promotes reductions in walking speed, functional parameters, and aerobic fitness in older, healthy adults. Journals of Gerontology—Series A Biological Sciences and Medical Sciences, 70(1), 91–96. 10.1093/gerona/glu123 PMC4342684 25122628

[eph70468-bib-0028] Collins, K. H. , Gui, C. , Ely, E. V. , Lenz, K. L. , Harris, C. A. , Guilak, F. , & Meyer, G. A. (2022). Leptin mediates the regulation of muscle mass and strength by adipose tissue. The Journal of Physiology, 600(16), 3795–3817. 10.1113/JP283034 PMC9378542 35844058

[eph70468-bib-0029] Dardevet, D. , Rémond, D. , Peyron, M. A. , Papet, I. , Savary‐Auzeloux, I. , & Mosoni, L. (2012). Muscle wasting and resistance of muscle anabolism: The “anabolic threshold concept” for adapted nutritional strategies during sarcopenia. The Scientific World Journal, 2012, 269531. 10.1100/2012/269531 PMC3541599 23326214

[eph70468-bib-0030] Delmonico, M. J. , Kostek, M. C. , Johns, J. , Hurley, B. F. , & Conway, J. M. (2008). Can dual energy X‐ray absorptiometry provide a valid assessment of changes in thigh muscle mass with strength training in older adults? European Journal of Clinical Nutrition, 62(12), 1372–1378. 10.1038/sj.ejcn.1602880 17684523

[eph70468-bib-0031] Deutz, N. E. P. , Pereira, S. L. , Hays, N. P. , Oliver, J. S. , Edens, N. K. , Evans, C. M. , & Wolfe, R. R. (2013). Effect of β‐hydroxy‐β‐methylbutyrate (HMB) on lean body mass during 10 days of bed rest in older adults. Clinical Nutrition, 32(5), 704–712. 10.1016/j.clnu.2013.02.011 23514626

[eph70468-bib-0032] Di Girolamo, F. G. , Fiotti, N. , Milanović, Z. , Situlin, R. , Mearelli, F. , Vinci, P. , Šimunič, B. , Pišot, R. , Narici, M. , & Biolo, G. (2021). The aging muscle in experimental bed rest: A systematic review and meta‐analysis. Frontiers in Nutrition, 8, 633987. 10.3389/fnut.2021.633987 PMC8371327 34422875

[eph70468-bib-0033] Dowling, L. , Lynch, D. H. , Batchek, D. , Sun, C. , Mark‐Wagstaff, C. , Jones, E. , Prochaska, M. , Huisingh‐Sheetz, M. , & Batsis, J. A. (2024). Nutrition interventions for body composition, physical function, cognition in hospitalized older adults: A systematic review of individuals 75 years and older. Journal of the American Geriatrics Society, 72(7), 2206–2218. 10.1111/jgs.18799 PMC11226365 38376064

[eph70468-bib-0034] Drummond, M. J. , Dickinson, J. M. , Fry, C. S. , Walker, D. K. , Gundermann, D. M. , Reidy, P. T. , Timmerman, K. L. , Markofski, M. M. , Paddon‐Jones, D. , Rasmussen, B. B. , Volpi, E. , & Bed, V. E. (2012). Bed rest impairs skeletal muscle amino acid transporter expression, mTORC1 signaling, and protein synthesis in response to essential amino acids in older adults. American Journal of Physiology‐Endocrinology and Metabolism, 302(9), 1113–1122. 10.1152/ajpendo.00603.2011 PMC3361979 22338078

[eph70468-bib-0035] Dulac, M. , Hajj‐Boutros, G. , Sonjak, V. , Faust, A. , Hussain, S. N. A. , Chevalier, S. , Dionne, I. J. , Morais, J. A. , & Gouspillou, G. (2025). A multimodal exercise countermeasure prevents the negative impact of head‐down tilt bed rest on muscle volume and mitochondrial health in older adults. The Journal of Physiology, 603(13), 3813–3836. 10.1113/JP285897 PMC12306415 38878232

[eph70468-bib-0036] Duncan, R. L. , & Turner, C. H. (1995). Mechanotransduction and the functional response of bone to mechanical strain. Calcified Tissue International, 57(5), 344–358. 10.1007/BF00302070 8564797

[eph70468-bib-0037] Eimori, K. , Endo, N. , Uchiyama, S. , Takahashi, Y. , Kawashima, H. , & Watanabe, K. (2016). Disrupted bone metabolism in long‐term bedridden patients. PLOS ONE, 11(6), e0156991. 10.1371/journal.pone.0156991 PMC4898699 27275738

[eph70468-bib-0038] Elam, C. , Hvid, L. G. , Christensen, U. , Kjær, M. , Magnusson, S. P. , Aagaard, P. , Bunketorp Käll, L. , & Suetta, C. (2022). Effects of age on muscle power, postural control and functional capacity after short‐term immobilization and retraining. Journal of Musculoskeletal & Neuronal Interactions, 22(4), 486–497. PMC9716293 36458386

[eph70468-bib-0039] English, K. L. , & Paddon‐Jones, D. (2010). Protecting muscle mass and function in older adults during bed rest. Current Opinion in Clinical Nutrition and Metabolic Care, 13(1), 34–39. 10.1097/MCO.0b013e328333aa66 PMC3276215 19898232

[eph70468-bib-0040] Erlandson, M. C. , Lorbergs, A. L. , Mathur, S. , & Cheung, A. M. (2016). Muscle analysis using pQCT, DXA and MRI. European Journal of Radiology, 85(8), 1505–1511. 10.1016/j.ejrad.2016.03.001 27005009

[eph70468-bib-0041] Ferrando, A. A. , Lane, H. W. , Stuart, C. A. , Davis‐Street, J. , & Wolfe, R. R. (1996). Prolonged bed rest decreases skeletal muscle and whole body protein synthesis. American Journal of Physiology‐Endocrinology and Metabolism, 270(4), E627–E633. 10.1152/ajpendo.1996.270.4.E627 8928769

[eph70468-bib-0042] Fiore, C. , Pennisi, P. , Ciffo, F. , Scebba, C. , Amico, A. , & Di Fazzio, S. (1999). Immobilization‐dependent bone collagen breakdown appears to increase with time: Evidence for a lack of a new bone equilibrium in response to reduced load during prolonged bed rest. Hormone and Metabolic Research, 31(1), 31–36. 10.1055/s-2007-978693 10077347

[eph70468-bib-0043] Franchi, M. V. , Raiteri, B. J. , Longo, S. , Sinha, S. , Narici, M. V. , & Csapo, R. (2018). Muscle architecture assessment: Strengths, shortcomings and new frontiers of in vivo imaging techniques. Ultrasound in Medicine and Biology, 44(12), 2492–2504. 10.1016/j.ultrasmedbio.2018.07.010 30185385

[eph70468-bib-0044] Frings‐Meuthen, P. , Boehme, G. , Liphardt, A.‐M. , Baecker, N. , Heer, M. , & Rittweger, J. (2013). Sclerostin and DKK1 levels during 14 and 21 days of bed rest in healthy young men. Journal of Musculoskeletal & Neuronal Interactions, 13(1), 45–52. 23445914

[eph70468-bib-0045] Fuchs, C. J. , Hermans, W. J. H. , van den Hurk, J. , Wiggins, C. J. , Widholm, P. , Dahlqvist Leinhard, O. , Veeraiah, P. , Wildberger, J. E. , Prompers, J. J. , & van Loon, L. J. C. (2025). Quantifying leg muscle disuse atrophy during bed rest using DXA, CT, and MRI. European Journal of Sport Science, 25(5), e12299. 10.1002/ejsc.12299 PMC11985322 40211056

[eph70468-bib-0046] Fuchs, C. J. , Kuipers, R. , Rombouts, J. A. , Brouwers, K. , Schrauwen‐Hinderling, V. B. , Wildberger, J. E. , Verdijk, L. B. , & van Loon, L. J. C. (2023). Thigh muscles are more susceptible to age‐related muscle loss when compared to lower leg and pelvic muscles. Experimental Gerontology, 175, 112159. 10.1016/j.exger.2023.112159 36967049

[eph70468-bib-0047] Gardner, R. L. , Harris, F. , Vittinghoff, E. , & Cummings, S. R. (2008). The risk of fracture following hospitalization in older women and men. Archives of Internal Medicine, 168(15), 1671–1677. 10.1001/archinte.168.15.1671 18695081

[eph70468-bib-0048] Gaudio, A. , Pennisi, P. , Bratengeier, C. , Torrisi, V. , Lindner, B. , Mangiafico, R. A. , Pulvirenti, I. , Hawa, G. , Tringali, G. , & Fiore, C. E. (2010). Increased sclerostin serum levels associated with bone formation and resorption markers in patients with immobilization‐induced bone loss. Journal of Clinical Endocrinology and Metabolism, 95(5), 2248–2253. 10.1210/jc.2010-0067 20305005

[eph70468-bib-0049] Glover, E. I. , Phillips, S. M. , Oates, B. R. , Tang, J. E. , Tarnopolsky, M. A. , Selby, A. , Smith, K. , & Rennie, M. J. (2008). Immobilization induces anabolic resistance in human myofibrillar protein synthesis with low and high dose amino acid infusion. The Journal of Physiology, 586(24), 6049–6061. 10.1113/jphysiol.2008.160333 PMC2655417 18955382

[eph70468-bib-0050] González‐Rocha, A. , Mendez‐Sanchez, L. , Ortíz‐Rodríguez, M. A. , & Denova‐Gutiérrez, E. (2022). Effect Of exercise on muscle mass, fat mass, bone mass, muscular strength and physical performance in community dwelling older adults: Systematic review and meta‐analysis. Aging and Disease, 13(5), 1421–1435. 10.14336/AD.2022.0215 PMC9466973 36186132

[eph70468-bib-0051] Hajj‐Boutros, G. , Sonjak, V. , Faust, A. , Hedge, E. , Mastrandrea, C. , Lagacé, J. C. , St‐Martin, P. , Naz Divsalar, D. , Sadeghian, F. , Chevalier, S. , Liu‐Ambrose, T. , Blaber, A. P. , Dionne, I. J. , Duchesne, S. , Hughson, R. , Kontulainen, S. , Theou, O. , & Morais, J. A. (2023). Impact of 14 days of bed rest in older adults and an exercise countermeasure on body composition, muscle strength, and cardiovascular function: Canadian space agency standard measures. Gerontology, 69(11), 1284–1294. 10.1159/000534063 PMC10634275 37717560

[eph70468-bib-0052] Hedge, E. T. , Patterson, C. A. , Mastrandrea, C. J. , Sonjak, V. , Hajj‐Boutros, G. , Faust, A. , Morais, J. A. , & Hughson, R. L. (2022). Implementation of exercise countermeasures during spaceflight and microgravity analogue studies: Developing countermeasure protocols for bedrest in older adults (BROA). Frontiers in Physiology, 13, 928313. 10.3389/fphys.2022.928313 PMC9395735 36017336

[eph70468-bib-0053] Herrmann, M. , Engelke, K. , Ebert, R. , Müller‐Deubert, S. , Rudert, M. , Ziouti, F. , Jundt, F. , Felsenberg, D. , & Jakob, F. (2020). Interactions between muscle and bone—where physics meets biology. Biomolecules, 10(3), 432. 10.3390/biom10030432 PMC7175139 32164381

[eph70468-bib-0054] Hirschfeld, H. P. , Kinsella, R. , & Duque, G. (2017). Osteosarcopenia: Where bone, muscle, and fat collide. Osteoporosis International, 28(10), 2781–2790. 10.1007/s00198-017-4151-8 28733716

[eph70468-bib-0055] Horber, F. F. , Thomi, F. , Casez, J. P. , Fonteille, J. , & Jaeger, P. (1992). Impact of hydration status on body composition as measured by dual energy X‐ray absorptiometry in normal volunteers and patients on haemodialysis*. The British Journal of Radiology, 65(778), 895–900. 10.1259/0007-1285-65-778-895 1422663

[eph70468-bib-0056] Inoue, M. , Tanaka, H. , Moriwake, T. , Oka, M. , Sekiguchi, C. , & Seino, Y. (2000). Altered biochemical markers of bone turnover in humans during 120 days of bed rest. Bone, 26(3), 281–286. 10.1016/s8756-3282(99)00282-3 10710002

[eph70468-bib-0057] Irimia, J. M. , Guerrero, M. , Rodriguez‐Miguelez, P. , Cadefau, J. A. , Tesch, P. A. , Cussó, R. , & Fernandez‐Gonzalo, R. (2017). Metabolic adaptations in skeletal muscle after 84 days of bed rest with and without concurrent flywheel resistance exercise. Journal of Applied Physiology, 122(1), 96–103. 10.1152/japplphysiol.00521.2016 27932674

[eph70468-bib-0058] Karsenty, G. , & Olson, E. N. (2016). Bone and muscle endocrine functions: Unexpected paradigms of inter‐organ communication. Cell, 164(6), 1248–1256. 10.1016/j.cell.2016.02.043 PMC4797632 26967290

[eph70468-bib-0059] Kazakia, G. J. , Tjong, W. , Nirody, J. A. , Burghardt, A. J. , Carballido‐Gamio, J. , Patsch, J. M. , Link, T. , Feeley, B. T. , & Benjamin Ma, C. (2014). The influence of disuse on bone microstructure and mechanics assessed by HR‐pQCT. Bone, 63, 132–140. 10.1016/j.bone.2014.02.014 PMC4041600 24603002

[eph70468-bib-0060] Kelleher, A. R. , Kimball, S. R. , Dennis, M. D. , Schilder, R. J. , & Jefferson, L. S. (2013). The mTORC1 signaling repressors REDD1/2 are rapidly induced and activation of p70S6K1 by leucine is defective in skeletal muscle of an immobilized rat hindlimb. American Journal of Physiology‐Endocrinology and Metabolism, 304, 229–236. 10.1152/ajpendo.00409.2012 PMC3543567 23193052

[eph70468-bib-0061] Kenny, H. C. , Rudwill, F. , Breen, L. , Salanova, M. , Blottner, D. , Heise, T. , Heer, M. , Blanc, S. , & O'Gorman, D. J. (2017). Bed rest and resistive vibration exercise unveil novel links between skeletal muscle mitochondrial function and insulin resistance. Diabetologia, 60(8), 1491–1501. 10.1007/s00125-017-4298-z 28500394

[eph70468-bib-0062] Kim, H. , Iwasaki, K. , Miyake, T. , Shiozawa, T. , Nozaki, S. , & Yajima, K. (2003). Changes in bone turnover markers during 14‐day 6° head‐down bed rest. Journal of Bone and Mineral Metabolism, 21(5), 311–315. 10.1007/s00774-003-0426-6 12928833

[eph70468-bib-0063] Kirk, B. , Feehan, J. , Lombardi, G. , & Duque, G. (2020). Muscle, bone, and fat crosstalk: The biological role of myokines, osteokines, and adipokines. Current Osteoporosis Reports, 18(4), 388–400. 10.1007/s11914-020-00599-y 32529456

[eph70468-bib-0064] Kirk, B. , Lombardi, G. , & Duque, G. (2025). Bone and muscle crosstalk in ageing and disease. Nature Reviews Endocrinology, 21(6), 375–390. 10.1038/s41574-025-01088-x 40011751

[eph70468-bib-0065] Kirk, B. , Prokopidis, K. , & Duque, G. (2021). Nutrients to mitigate osteosarcopenia: The role of protein, vitamin D and calcium. Current Opinion in Clinical Nutrition and Metabolic Care, 24(1), 25–32. 10.1097/MCO.0000000000000711 33148944

[eph70468-bib-0066] Klein‐Nulend, J. , Bakker, A. D. , Bacabac, R. G. , Vatsa, A. , & Weinbaum, S. (2013). Mechanosensation and transduction in osteocytes. Bone, 54(2), 182–190. 10.1016/j.bone.2012.10.013 23085083

[eph70468-bib-0067] Kortebein, P. , Ferrando, A. , Lombeida, J. , Wolfe, R. , & Evans, W. J. (2007). Effect of 10 days of bed rest on skeletal muscle in healthy older adults. The Journal of the American Medical Association, 297(16), 1769. 10.1001/jama.297.16.1772-b 17456818

[eph70468-bib-0068] Kortebein, P. , Symons, T. B. , Ferrando, A. , Paddon‐Jones, D. , Ronsen, O. , Protas, E. , Conger, S. , Lombeida, J. , Wolfe, R. , & Evans, W. J. (2008). Functional impact of 10 days of bed rest in healthy older adults. The Journals of Gerontology Series A: Biological Sciences and Medical Sciences, 63(10), 1076–1081. 10.1093/gerona/63.10.1076 18948558

[eph70468-bib-0069] Krause, M. P. , Milne, K. J. , & Hawke, T. J. (2019). Adiponectin—consideration for its role in skeletal muscle health. International Journal of Molecular Sciences, 20(7), 1528. 10.3390/ijms20071528 PMC6480271 30934678

[eph70468-bib-0070] Laharrague, P. , Fontanilles, A. M. , Tkaczuk, J. , Corberand, J. X. , Pénicaud, L. , & Casteilla, L. (2000). Inflammatory/haematopoietic cytokine production by human bone marrow adipocytes. European Cytokine Network, 11(4), 634–639. 11125307

[eph70468-bib-0071] LeBlanc, A. D. , Spector, E. R. , Evans, H. J. , & Sibonga, J. D. (2007). Skeletal responses to space flight and the bed rest analog: A review. Journal of Musculoskeletal & Neuronal Interactions, 7(1), 33–47. 17396004

[eph70468-bib-0072] Li, G. B. , Zhang, L. , Wang, D. E. , AIQudsy, L. , Jiang, J. X. , Xu, H. Y. , & Shang, P. (2019). Muscle‐bone crosstalk and potential therapies for sarco‐osteoporosis. Journal of Cellular Biochemistry, 120(9), 14262–14273. 10.1002/jcb.28946 PMC7331460 31106446

[eph70468-bib-0073] Li, Q. , Wu, Y. , & Kang, N. (2018). Marrow adipose tissue: Its origin, function, and regulation in bone remodeling and regeneration. Stem Cells International, 2018, 7098456. 10.1155/2018/7098456 PMC6000863 29955232

[eph70468-bib-0074] Liu, T. , Melkus, G. , Ramsay, T. , Sheikh, A. , Laneuville, O. , & Trudel, G. (2021). Bone marrow reconversion with reambulation: A prospective clinical trial. Investigative Radiology, 56(4), 215–223. 10.1097/RLI.0000000000000730 33038096

[eph70468-bib-0075] MacNeil, J. A. , & Boyd, S. K. (2007). Accuracy of high‐resolution peripheral quantitative computed tomography for measurement of bone quality. Medical Engineering and Physics, 29(10), 1096–1105. 10.1016/j.medengphy.2006.11.002 17229586

[eph70468-bib-0076] Manini, T. M. , Visser, M. , Won‐Park, S. , Patel, K. V. , Strotmeyer, E. S. , Chen, H. , Goodpaster, B. , De Rekeneire, N. , Newman, A. B. , Simonsick, E. M. , Kritchevsky, S. B. , Ryder, K. , Schwartz, A. V. , & Harris, T. B. (2007). Knee extension strength cutpoints for maintaining mobility. Journal of the American Geriatrics Society, 55(3), 451–457. 10.1111/j.1532-5415.2007.01087.x 17341251

[eph70468-bib-0077] McGrath, E. R. , Frings‐Meuthen, P. , Sibonga, J. , Heer, M. , Clement, G. R. , Mulder, E. , Smith, S. M. , & Zwart, S. R. (2022). Bone metabolism during strict head‐down tilt bed rest and exposure to elevated levels of ambient CO2. Nature Partner Journal Microgravity, 8(1), 57. 10.1038/s41526-022-00245-0 PMC9758179 36526672

[eph70468-bib-0078] Mera, P. , Laue, K. , Ferron, M. , Confavreux, C. , Wei, J. , Galán‐Díez, M. , Lacampagne, A. , Mitchell, S. J. , Mattison, J. A. , Chen, Y. , Bacchetta, J. , Szulc, P. , Kitsis, R. N. , De Cabo, R. , Friedman, R. A. , Torsitano, C. , McGraw, T. E. , Puchowicz, M. , Kurland, I. , & Karsenty, G. (2016). Osteocalcin signaling in myofibers is necessary and sufficient for optimum adaptation to exercise. Cell Metabolism, 23(6), 1078–1092. 10.1016/j.cmet.2016.05.004 PMC4910629 27304508

[eph70468-bib-0079] Miokovic, T. , Armbrecht, G. , Gast, U. , Rawer, R. , Roth, H. J. , Runge, M. , Felsenberg, D. , & Belavý, D. L. (2014). Muscle atrophy, pain, and damage in bed rest reduced by resistive (Vibration) exercise. Medicine and Science in Sports and Exercise, 46(8), 1506–1516. 10.1249/MSS.0000000000000279 24561811

[eph70468-bib-0080] Monti, E. , Reggiani, C. , Franchi, M. V. , Toniolo, L. , Sandri, M. , Armani, A. , Zampieri, S. , Giacomello, E. , Sarto, F. , Sirago, G. , Murgia, M. , Nogara, L. , Marcucci, L. , Ciciliot, S. , Šimunic, B. , Pišot, R. , & Narici, M. V. (2021). Neuromuscular junction instability and altered intracellular calcium handling as early determinants of force loss during unloading in humans. The Journal of Physiology, 599(12), 3037–3061. 10.1113/JP281365 PMC8359852 33881176

[eph70468-bib-0081] Nana, A. , Slater, G. J. , Hopkins, W. G. , & Burke, L. M. (2012). Effects of daily activities on dual‐energy X‐ray absorptiometry measurements of body composition in active people. Medicine and Science in Sports and Exercise, 44(1), 180–189. 10.1249/MSS.0b013e318228b60e 22179140

[eph70468-bib-0082] Nunes, E. A. , Stokes, T. , McKendry, J. , Currier, B. S. , & Phillips, S. M. (2022). Disuse‐induced skeletal muscle atrophy in disease and non‐disease states in humans: Mechanisms, prevention, and recovery strategies. American Journal of Physiology—Cell Physiology, 322(6), C1068–C1084. 10.1152/ajpcell.00425.2021 35476500

[eph70468-bib-0083] Oranger, A. , Storlino, G. , Dicarlo, M. , Zerlotin, R. , Pignataro, P. , Sanesi, L. , Narici, M. , Pišot, R. , Simunič, B. , Colaianni, G. , Grano, M. , & Colucci, S. (2023). Impact of 10‐day bed rest on serum levels of irisin and markers of musculoskeletal metabolism. The Federation of American Societies for Experimental Biology Journal, 37(1), e22668. 10.1096/fj.202201005RR PMC13281841 36475382

[eph70468-bib-0084] Orchard, T. S. , Pan, X. , Cheek, F. , Ing, S. W. , & Jackson, R. D. (2012). A systematic review of omega‐3 fatty acids and osteoporosis. British Journal of Nutrition, 107(S2), S253–S260. 10.1017/S0007114512001638 PMC3899785 22591899

[eph70468-bib-0085] O'Reilly, C. L. , Bodine, S. C. , & Miller, B. F. (2025). Current limitations and future opportunities of tracer studies of muscle ageing. The Journal of Physiology, 603(1), 7–15. 10.1113/JP285616 PMC11150331 38051758

[eph70468-bib-0086] Paddon‐Jones, D. , Sheffield‐Moore, M. , Urban, R. J. , Sanford, A. P. , Aarsland, A. , Wolfe, R. R. , & Ferrando, A. A. (2004). Essential amino acid and carbohydrate supplementation ameliorates muscle protein loss in humans during 28 days bedrest. Journal of Clinical Endocrinology and Metabolism, 89(9), 4351–4358. 10.1210/jc.2003-032159 15356032

[eph70468-bib-0087] Pišot, R. , Marusic, U. , Biolo, G. , Mazzucco, S. , Lazzer, S. , Grassi, B. , Reggiani, C. , Toniolo, L. , Di Prampero, P. E. , Passaro, A. , Narici, M. , Mohammed, S. , Rittweger, J. , Gasparini, M. , Blenkuŝ, M. G. , & Šimuniĉ, B. (2016). Greater loss in muscle mass and function but smaller metabolic alterations in older compared with younger men following 2 week of bed rest and recovery. Journal of Applied Physiology, 120(8), 922–929. 10.1152/japplphysiol.00858.2015 26823343

[eph70468-bib-0088] Prokopidis, K. , Giannos, P. , Kirwan, R. , Ispoglou, T. , Galli, F. , Witard, O. C. , Triantafyllidis, K. K. , Kechagias, K. S. , Morwani‐Mangnani, J. , Ticinesi, A. , & Isanejad, M. (2023). Impact of probiotics on muscle mass, muscle strength and lean mass: A systematic review and meta‐analysis of randomized controlled trials. Journal of Cachexia, Sarcopenia and Muscle, 14(1), 30–44. 10.1002/jcsm.13132 PMC9891957 36414567

[eph70468-bib-0089] Prokopidis, K. , Morgan, P. T. , Deane, C. S. , Witard, O. C. , & Church, D. D. (2025). The effect of bed rest, unilateral limb immobilization and head‐down tilt on muscle protein synthesis: A systematic review and meta‐analysis. Experimental Physiology. Advance online publication. 10.1113/EP092474 PMC13394532 41166041

[eph70468-bib-0090] Prokopidis, K. , Reyes, J. M. , & Duque, G. (2026). Effects of bed rest and immobilization on intramuscular and intermuscular adipose tissue: A systematic review. Experimental Physiology. Advance online publication. 10.1113/EP093306 PMC13394330 41729965

[eph70468-bib-0091] Qian, X. X. , Chen, Z. , Fong, D. Y. T. , Ho, M. , & Chau, P. H. (2022). Post‐hospital falls incidence and risk factors among older adults: A systematic review and meta‐analysis. Age and Ageing, 51(1), afab209. 10.1093/ageing/afab209 34718373

[eph70468-bib-0092] Ramnemark, A. , Nyberg, L. , Borssén, B. , Olsson, T. , & Gustafson, Y. (1998). Fractures after stroke. Osteoporosis International, 8(1), 92–95. 10.1007/s001980050053 9692083

[eph70468-bib-0093] Rejc, E. , Floreani, M. , Taboga, P. , Botter, A. , Toniolo, L. , Cancellara, L. , Narici, M. , Šimunič, B. , Pišot, R. , Biolo, G. , Passaro, A. , Rittweger, J. , Reggiani, C. , & Lazzer, S. (2018). Loss of maximal explosive power of lower limbs after 2 weeks of disuse and incomplete recovery after retraining in older adults. The Journal of Physiology, 596(4), 647–665. 10.1113/JP274772 PMC5813607 29266264

[eph70468-bib-0094] Rittweger, J. , Beller, G. , Armbrecht, G. , Mulder, E. , Buehring, B. , Gast, U. , Dimeo, F. , Schubert, H. , de Haan, A. , Stegeman, D. F. , Schiessl, H. , & Felsenberg, D. (2010). Prevention of bone loss during 56 days of strict bed rest by side‐alternating resistive vibration exercise. Bone, 46(1), 137–147. 10.1016/j.bone.2009.08.051 19732856

[eph70468-bib-0095] Rittweger, J. , Simunic, B. , Bilancio, G. , Gaspare De Santo, N. , Cirillo, M. , Biolo, G. , Pisot, R. , Eiken, O. , Mekjavic, I. B. , & Narici, M. (2009). Bone loss in the lower leg during 35 days of bed rest is predominantly from the cortical compartment. Bone, 44(4), 612–618. 10.1016/j.bone.2009.01.001 19168165

[eph70468-bib-0096] Sanesi, L. , Storlino, G. , Dicarlo, M. , Oranger, A. , Zerlotin, R. , Pignataro, P. , Suriano, C. , Guida, G. , Grano, M. , Colaianni, G. , & Colucci, S. C. (2023). Time‐dependent unloading effects on muscle and bone and involvement of FNDC5/irisin axis. Nature Partner Journal Microgravity, 9(1), 4. 10.1038/s41526-023-00251-w PMC9852594 36658231

[eph70468-bib-0097] Sarfati, M. , Chapurlat, R. , Dufour, A. B. , Sornay‐Rendu, E. , Merle, B. , Boyd, S. K. , Whittier, D. E. , Hanley, D. A. , Goltzman, D. , Szulc, P. , Wong, A. K. O. , Lespessailles, E. , Khosla, S. , Ferrari, S. , Biver, E. , Ohlsson, C. , Lorentzon, M. , Mellström, D. , Nethander, M. , & Bouxsein, M. L. (2024). Short‐term risk of fracture is increased by deficits in cortical and trabecular bone microarchitecture independent of DXA BMD and FRAX: Bone Microarchitecture International Consortium (BoMIC) prospective cohorts. Journal of Bone and Mineral Research, 39(11), 1574–1583. 10.1093/jbmr/zjae143 PMC11523184 39236248

[eph70468-bib-0098] Schnyder, S. , & Handschin, C. (2015). Skeletal muscle as an endocrine organ: PGC‐1α, myokines and exercise. Bone, 80, 115–125. 10.1016/j.bone.2015.02.008 PMC4657151 26453501

[eph70468-bib-0099] Shackelford, L. C. , Leblanc, A. D. , Driscoll, T. B. , Evans, H. J. , Rianon, N. J. , Smith, S. M. , Spector, E. , Feeback, D. L. , & Lai, D. (2004). Resistance exercise as a countermeasure to disuse‐induced bone loss. Journal of Applied Physiology, 97, 119–129. 10.1152/japplphysiol.00741.2003 15220316

[eph70468-bib-0100] Shur, N. F. , Simpson, E. J. , Crossland, H. , Constantin, D. , Cordon, S. M. , Constantin‐Teodosiu, D. , Stephens, F. B. , Brook, M. S. , Atherton, P. J. , Smith, K. , Wilkinson, D. J. , Mougin, O. E. , Bradley, C. , Macdonald, I. A. , & Greenhaff, P. L. (2024). Bed‐rest and exercise remobilization: Concurrent adaptations in muscle glucose and protein metabolism. Journal of Cachexia, Sarcopenia and Muscle, 15(2), 603–614. 10.1002/jcsm.13431 PMC10995277 38343303

[eph70468-bib-0101] Smith, G. I. , Atherton, P. , Reeds, D. N. , Mohammed, B. S. , Rankin, D. , Rennie, M. J. , & Mittendorfer, B. (2011). Dietary omega‐3 fatty acid supplementation increases the rate of muscle protein synthesis in older adults: A randomized controlled trial. American Journal of Clinical Nutrition, 93(2), 402–412. 10.3945/ajcn.110.005611 PMC3021432 21159787

[eph70468-bib-0102] Smith, S. M. , Zwart, S. R. , Heer, M. A. , Baecker, N. , Evans, H. J. , Feiveson, A. H. , Shackelford, L. C. , & LeBlanc, A. D. (2009). Effects of artificial gravity during bed rest on bone metabolism in humans. Journal of Applied Physiology, 107(1), 47–53. 10.1152/japplphysiol.91134.2008 PMC2711792 19074572

[eph70468-bib-0103] Sornay‐Rendu, E. , Boutroy, S. , Duboeuf, F. , & Chapurlat, R. D. (2017). Bone microarchitecture assessed by HR‐pQCT as predictor of fracture risk in postmenopausal women: The OFELY study. Journal of Bone and Mineral Research, 32(6), 1243–1251. 10.1002/jbmr.3105 28276092

[eph70468-bib-0104] Spatz, J. M. , Fields, E. E. , Yu, E. W. , Divieti Pajevic, P. , Bouxsein, M. L. , Sibonga, J. D. , Zwart, S. R. , & Smith, S. M. (2012). Serum sclerostin increases in healthy adult men during bed rest. Journal of Clinical Endocrinology and Metabolism, 97(9), E1736–E1740. 10.1210/jc.2012-1579 PMC3431567 22767636

[eph70468-bib-0105] Spatz, J. M. , Wein, M. N. , Gooi, J. H. , Qu, Y. , Garr, J. L. , Liu, S. , Barry, K. J. , Uda, Y. , Lai, F. , Dedic, C. , Balcells‐Camps, M. , Kronenberg, H. M. , Babij, P. , & Pajevic, P. D. (2015). The Wnt inhibitor sclerostin is up‐regulated by mechanical unloading in osteocytes in vitro. Journal of Biological Chemistry, 290(27), 16744–16758. 10.1074/jbc.M114.628313 PMC4505423 25953900

[eph70468-bib-0106] Standley, R. A. , Distefano, G. , Pereira, S. L. , Tian, M. , Kelly, O. J. , Coen, P. M. , Deutz, N. E. P. , Wolfe, R. R. , & Goodpaster, B. H. (2017). Effects of‐hydroxy‐methylbutyrate on skeletal muscle mitochondrial content and dynamics, and lipids after 10 days of bed rest in older adults. Journal of Applied Physiology, 123, 1092–1100. 10.1152/japplphysiol.00192.2017 28705993

[eph70468-bib-0107] Suetta, C. , Frandsen, U. , Jensen, L. , Jensen, M. M. , Jespersen, J. G. , Hvid, L. G. , Bayer, M. , Petersson, S. J. , Schrøder, H. D. , Andersen, J. L. , Heinemeier, K. M. , Aagaard, P. , Schjerling, P. , & Kjaer, M. (2012). Aging affects the transcriptional regulation of human skeletal muscle disuse atrophy. PLOS ONE, 7(12), e51238. 10.1371/journal.pone.0051238 PMC3526599 23284670

[eph70468-bib-0108] Sui, H. , Dou, J. , Shi, B. , & Cheng, X. (2024). The reciprocity of skeletal muscle and bone: An evolving view from mechanical coupling, secretory crosstalk to stem cell exchange. Frontiers in Physiology, 15, 1349253. 10.3389/fphys.2024.1349253 PMC10949226 38505709

[eph70468-bib-0109] Symons, T. B. , Sheffield‐Moore, M. , Chinkes, D. L. , Ferrando, A. A. , & Paddon‐Jones, D. (2009). Artificial gravity maintains skeletal muscle protein synthesis during 21 days of simulated microgravity. Journal of Applied Physiology, 107, 34–38. 10.1152/japplphysiol.91137.2008 PMC2711793 19390002

[eph70468-bib-0110] Tanner, R. E. , Brunker, L. B. , Agergaard, J. , Barrows, K. M. , Briggs, R. A. , Kwon, O. S. , Young, L. M. , Hopkins, P. N. , Volpi, E. , Marcus, R. L. , Lastayo, P. C. , & Drummond, M. J. (2015). Age‐related differences in lean mass, protein synthesis and skeletal muscle markers of proteolysis after bed rest and exercise rehabilitation. The Journal of Physiology, 593(18), 4259–4273. 10.1113/JP270699 PMC4594296 26173027

[eph70468-bib-0111] Ticinesi, A. , Lauretani, F. , Milani, C. , Nouvenne, A. , Tana, C. , Del Rio, D. , Maggio, M. , Ventura, M. , & Meschi, T. (2017). Aging gut microbiota at the cross‐road between nutrition, physical frailty, and sarcopenia: Is there a gut–muscle axis? Nutrients, 9(12), 1303. 10.3390/nu9121303 PMC5748753 29189738

[eph70468-bib-0112] Trappe, S. , Trappe, T. , Gallagher, P. , Harber, M. , Alkner, B. , & Tesch, P. (2004). Human single muscle fibre function with 84 day bed‐rest and resistance exercise. The Journal of Physiology, 557(2), 501–513. 10.1113/jphysiol.2004.062166 PMC1665105 15064323

[eph70468-bib-0113] Trudel, G. , Coletta, E. , Cameron, I. , Belavý, D. L. , Lecompte, M. , Armbrecht, G. , Felsenberg, D. , & Uhthoff, H. K. (2012). Resistive exercises, with or without whole body vibration, prevent vertebral marrow fat accumulation during 60 days of head‐down tilt bed rest in men. Journal of Applied Physiology, 112, 1824–1831. 10.1152/japplphysiol.00029.2012 22442031

[eph70468-bib-0114] Trudel, G. , Payne, M. , Mädler, B. , Ramachandran, N. , Lecompte, M. , Wade, C. , Biolo, G. , Blanc, S. , Hughson, R. , Bear, L. , Uhthoff, H. K. , & dler, M. B. (2009). Bone marrow fat accumulation after 60 days of bed rest persisted 1 year after activities were resumed along with hemopoietic stimulation: The women international space simulation for exploration study. Journal of Applied Physiology, 107, 540–548. 10.1152/japplphysiol.91530.2008 19478189

[eph70468-bib-0115] Wall, B. T. , Dirks, M. L. , Snijders, T. , Senden, J. M. G. , Dolmans, J. , & Van Loon, L. J. C. (2014). Substantial skeletal muscle loss occurs during only 5 days of disuse. Acta Physiologica, 210(3), 600–611. 10.1111/apha.12190 24168489

[eph70468-bib-0116] Wang, H. , Zheng, X. , Zhang, Y. , Huang, J. , Zhou, W. , Li, X. , Tian, H. , Wang, B. , Xing, D. , Fu, W. , Chen, T. , Wang, X. , Zhang, X. , & Wu, A. (2021). The endocrine role of bone: Novel functions of bone‐derived cytokines. Biochemical Pharmacology, 183, 114308. 10.1016/j.bcp.2020.114308 33137323

[eph70468-bib-0117] Watanabe, Y. , Ohshima, H. , Mizuno, K. , Sekiguchi, C. , Fukunaga, M. , Kohri, K. , Rittweger, J. , Felsenberg, D. , Matsumoto, T. , & Nakamura, T. (2004). Intravenous pamidronate prevents femoral bone loss and renal stone formation during 90‐day bed rest. Journal of Bone and Mineral Research, 19(11), 1771–1778. 10.1359/JBMR.040811 15476576

[eph70468-bib-0118] Yang, C. , Chen, J. , Wu, F. , Li, J. , Liang, P. , Zhang, H. , Wang, H. , Li, Y. , Wan, Y. , Qin, L. , Liang, K. S. , Dai, Z. , & Li, Y. (2014). Effects of 60‐day head‐down bed rest on osteocalcin, glycolipid metabolism and their association with or without resistance training. Clinical Endocrinology, 81(5), 671–678. 10.1111/cen.12535 24975467

[eph70468-bib-0119] Zerwekh, J. E. , Ruml, L. A. , Gottschalk, F. , & Pak, C. Y. C. (1998). The effects of twelve weeks of bed rest on bone histology, biochemical markers of bone turnover, and calcium homeostasis in eleven normal subjects. Journal of Bone and Mineral Research, 13(10), 1594–1601. 10.1359/jbmr.1998.13.10.1594 9783548

[eph70468-bib-0120] Zhou, H. , Trudel, G. , Alexeev, K. , Thomas, J. , Laneuville, O. , Haodong Zhou, X. , Trudel, G. , Alexeev, K. , Thomas, J. , Laneuville, O. , & Hyper, L. O. (2020). Hyperplasia and accelerated hypertrophy of marrow adipocytes with knee immobilization were sustained despite remobilization. Journal of Applied Physiology, 129(4), 701–708. 10.1152/japplphysiol.00539.2020 32853104

